# Influence of gamma irradiation on physical, structural, and optical properties of Dy^3+^ doped barium bismuth borate sodium glasses

**DOI:** 10.1038/s41598-025-31194-9

**Published:** 2025-12-06

**Authors:** Meher Taj S, M. R. Ambika, C. Devaraja, Sudha D. Kamath, K. R. Vighnesh, A. S. Bennal, Utpal Deka

**Affiliations:** 1 Department of Physics, Manipal Institute of Technology Bengaluru, Manipal Academy of Higher Education, Manipal, Karnataka 576104 India; 2 Department of Physics, M S Ramaiah Institute of Technology, MSR Nagar, Bengaluru, 560054 India; 3 Department of Physics, Manipal Institute of Technology, Manipal Academy of Higher Education, Manipal, Karnataka 576104 India; 4 Department of Physics, Karnatak University , Dharwad, Karnataka 580003 India; 5 Department of Physics, Sikkim Manipal Institute of Technology, Sikkim Manipal University (SMU), Majitar, India

**Keywords:** Barium bismuth borate glasses, Dysprosium, FTIR and Raman analysis, UV–VIS–NIR, PL, Chemistry, Materials science, Optics and photonics, Physics

## Abstract

Barium bismuth borate sodium (BBBNDy) glasses doped with Dy_2_O_3_ (0–0.8 mol%, x = 0.2 mol%) were prepared via the melt quenching approach and examined under gamma irradiation. XRD confirmed the amorphous nature, while density measurements (4.3387–4.4743 g/cm^3^) revealed composition and irradiation-dependent variations. FTIR and Raman studies indicated Dy_2_O_3_-induced network modification through BO_3_/BO_4_ and BiO_3_/BiO_6_ units, with minimal structural degradation under irradiation. Optical studies revealed tunable band gaps and low Urbach energies (0.1752–0.2266 eV) reflecting good structural order. Photoluminescence spectra exhibited a strong yellow emission at 575 nm (^4^F_9/2_ → ^6^H_13/2_), with optimal intensity for BBBNDy3 glass. Gamma irradiation induced PL quenching at 575 nm, with intensity reduced by to 66.6% at 5 kGy and 87.2% at 15 kGy; still, Dy^3+^ emission remains detectable. These results highlight the dual role of Dy^3+^ ions as structural stabilisers and efficient luminescent activators, identifying BBBNDy3 glasses as a strong candidate for photonic devices operating in radiation environments.

## Introduction

Borate glasses, owing to the glass-forming ability of B_2_O_3_ (boric acid), offer structural flexibility, wide optical transparency, reduced melting temperatures, enhanced thermal/mechanical endurance, and strong rare-earth (RE) solubility^1–6^, making them attractive for solid-state lighting, fiber amplifiers, and radiation shielding applications^1–3,7,8^. Their high boron content provides a large neutron absorption cross section, and their effective atomic number (Z_eff_ = 7.42) is close to that of human tissue, making them relevant for clinical radiation protection uses such as dosimeters and implants^9^. The borate glass structure comprises both trigonal (BO_3_) and tetrahedral (BO_4_) structural units, forming complex arrangements such as boroxyl rings and various cyclic units of borate groups, varying from diborate to pentaborate configurations^4,8,10–15^. However, the high phonon energy of B_2_O_3_ (~ 1300 cm^−1^) promotes non-radiative losses, which can be mitigated by incorporating heavy metal oxides (HMOs) like Bismuth oxide (Bi_2_O_3_) and Barium oxide (BaO), enhancing fluorescence efficiency^4,7,16^ and improving shielding performance due to their high atomic numbers and densities^8,10^. Bi_2_O_3_ contributes BiO_3_, and BiO_6_ units, enhancing chemical durability and optical density^4,7,11^, while BaO promotes NBO formation, restructures the network, and improves thermal and optical response^8,17–22^. Additionally, Sodium oxide (Na_2_O) serves as a flux to lower melting temperatures, encourages BO_3_ to BO_4_ transformation, improving chemical resistance and tunability of physical properties^23–26^. Borate glasses exhibit high rare-earth (RE) ion solubility, making them suitable hosts for photonic and radiation shielding applications^27,28^. Unlike conventional shielding materials like concrete and polymers, RE-doped borate glasses offer transparency, chemical stability, and radiation tolerance. Among them, Dysprosium oxide(Dy_2_O_3_)-doped glasses stand out for their characteristic emissions in the blue (^4^F_9/2_ → ^6^H_15/2_), yellow (^4^F_9/2_ → ^6^H_13/2_), and weak red (^4^F_9/2_ → ^6^H_11/2_) regions, as well as infrared emissions at 1.32 μm (^6^H_9/2_ → ^6^H_15/2_) making it suitable for energy-efficient lighting display panels and optical communication systems^29–33^. Additionally, Dy^3+^ luminescence is highly sensitive to local structural features, including symmetry distortions, coordination sites, and the NBO content, which can be tailored via compositional design and irradiation^2,11,25,26,34,35^.

Gamma irradiation, a powerful form of ionizing radiation, significantly alters the structural and optical properties of glass materials. When high-energy gamma photons penetrate a glass network, they excite and displace electrons from their ground state, create defects such as oxygen vacancies and non-bridging oxygen atoms (NBO), and multivalent cationic sites^1,10^, disrupting the network bonds, compaction, and the formation of color centers through electron or hole trapping^1^. Consequently, studies involving borate, silicate, and phosphate-based glasses have revealed that gamma irradiation impacts the structure, particularly in borate glasses, transformations between BO_4_ and BO_3_ groups, and increased NBO content^1,36^. Studies by Marzouk and Ghoneim^16,37^ have confirmed these changes, showing only mild alterations in the intensity, shifts in vibrational bands, and the emergence of new features of the FTIR bands following gamma irradiation of glass ceramic doped with REE (rare earth elements), mainly associated with the variations in the bond geometry, such as angles and lengths. Gamma ray exposure also affects the dielectric characteristics and mass density of lithium borate-based glasses due to enhanced polarizability introduced by NBO formation and compaction of the network^1^. Furthermore, gamma irradiation narrows the optical energy band gap in glass materials, resulting in defect levels within the band gap region and absorption centers decreasing transparency in the UV–visible region^2,4^. Despite these structural modifications, most glasses subjected to high-dose gamma irradiation (up to several Mrads) continue to exhibit an amorphous nature, demonstrating their structural resilience and potential for shielding against ionizing radiation, in nuclear reactors, space missions, and medical facilities^1,38^.

Unlike traditional shielding materials like concrete, polymers, or lead-based materials, glasses offer transparency, chemical stability, ease of fabrication, and tunable composition^1,2,9^, making them an attractive alternative for advanced shielding applications.

The current study focuses on barium bismuth sodium borate glasses (BBBNDy), wherein BaO is systematically replaced with varying concentrations of Dy_2_O_3_ (0–0.8 mol%). The aim is to comprehensively explore the influence of Dy_2_O_3_ incorporation on the glass systems physical parameters (e.g., density), structural features (via FTIR and Raman technique), and optical behavior (UV–Vis–NIR absorption, optical band gaps, and luminescence) of the glass system before and after gamma exposure (5, 10, and 15 kGy). Among all the prepared glass compositions, the one doped with 0.6 mol% Dy_2_O_3_ (BBBNDy3) demonstrated the most intense photoluminescence emission before gamma irradiation. Only this optimized sample, as doped and an undoped sample, was selected for gamma irradiation studies. This focused approach enables a clearer understanding of how gamma dose affects the structural, optical, and physical traits of BBBNDy glass systems.

## Experimental techniques

### Glass synthesis

A series of glasses with the formula 55B_2_O_3_–(20 − x) BaO–10Na_2_O–15Bi_2_O_3_–xDy_2_O_3_, (x = 0, 0.2, 0.4, 0.6, and 0.8 mol%), was synthesized via the melt-quenching process. High-purity raw materials, including Boric acid (H_3_BO_3_, 99.5%), Barium carbonate (BaCO_3_, 99.9%), Sodium carbonate (Na_2_CO_3_, 99.9%), Bismuth oxide (Bi_2_O_3_, 99.9%), and Dysprosium oxide (Dy_2_O_3_, 99.9%), were precisely weighed according to the batch matrix using a digital balance. The weighed powders were then finely ground for 30 min to ensure uniform mixing.

**Fig. 1:**
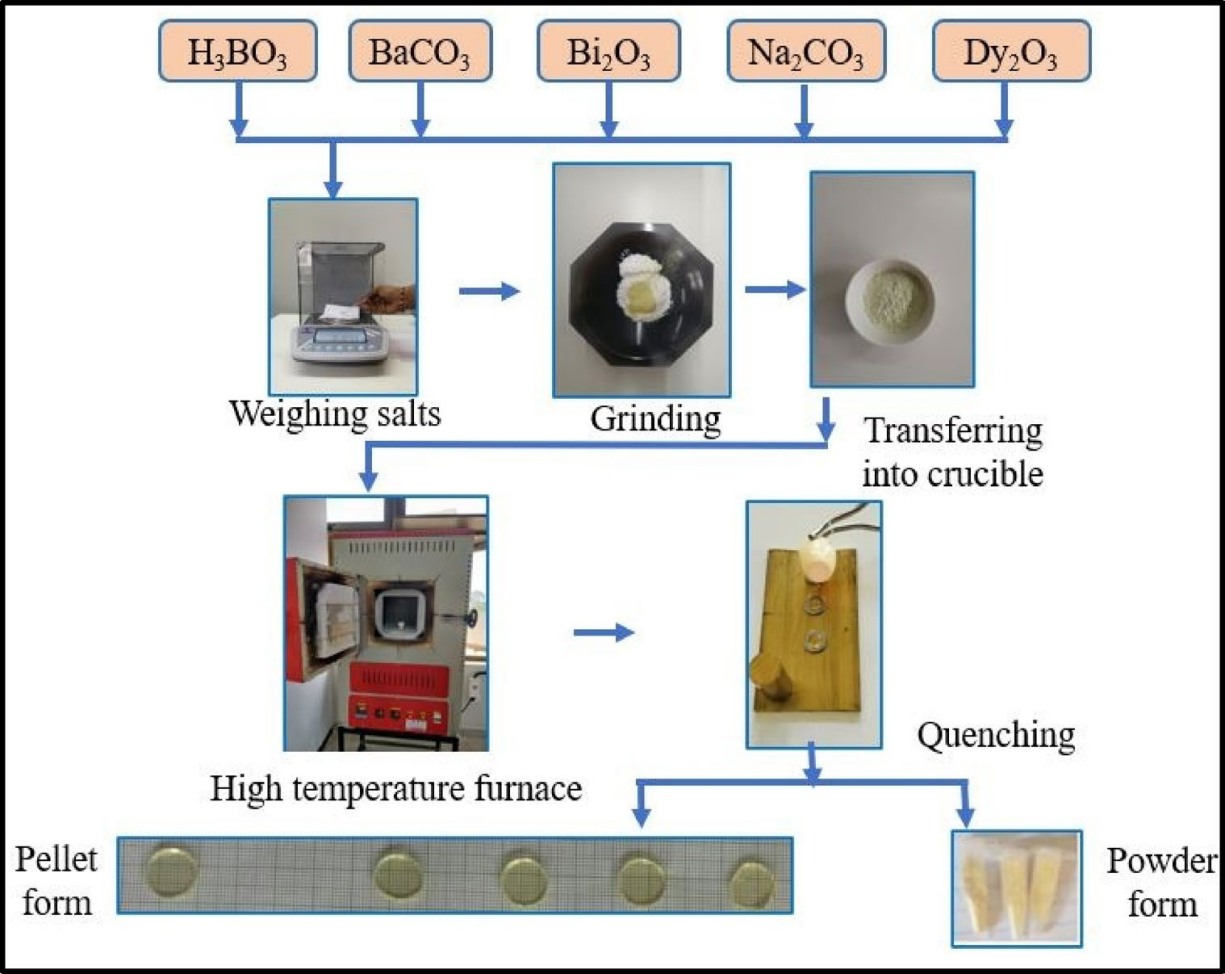
Schematic representation of the synthesis process for BBBNDy glass samples.

Following homogenization, the powder mixture was loaded into a porcelain crucible and heated from room temperature to 1050 °C at 10 °C per minute in an electric furnace. Upon reaching 1050 °C, the melt was held for 20 min and stirred to enhance uniformity, followed by an additional 10-min hold to ensure complete homogenization before quenching. The melt was poured into steel rings placed on a brass plate to obtain pellet-shaped samples at room temperature. Immediately after pouring, a brass weight was placed over the melt within the rings to minimize the thermal stress, ensuring the formation of stable glass pellets. Before evaluating the properties, a thickness of approximately 1–2 mm was achieved by polishing the glass samples with graded emery papers of varying grit sizes (320, 600, 800, 1000, 1200, and 1500) to achieve a smooth and uniform surface. Table 1 provides details on sample codes, compositions, and molecular weight (M_w_) of the BBBNDy series. The prepared glass samples exhibited a distinct yellow coloration and good transparency, as displayed in Fig. 1, along with the synthesis flowchart.

**Table 1:** Composition, Molecular weight of BBBNDy glasses.

Sample code	B_2_O_3_ (mol%)	Bi_2_O_3_ (mol%)	BaO (mol%)	Na_2_O (mol%)	Dy_2_O_3_ (mol%)	M_w_ (g/mol)
BBBNDy0	55	15	20	10	0	187.9760
BBBNDy1	55	15	19.8	10	0.2	188.3273
BBBNDy2	55	15	19.6	10	0.4	188.6786
BBBNDy3	55	15	19.4	10	0.6	189.0299
BBBNDy4	55	15	19.2	10	0.8	189.3812

### Material characterization methods

X-ray diffraction (XRD) analysis was carried out using a Rigaku Miniflex 600 benchtop diffractometer confirmed the non-crystalline nature of the BBBNDy glasses. It operates at 40 kV, 15 mA copper X-ray tube, generating Cu-Kα radiation (λ = 1.5406 Å). It supports scanning speeds from 0.01 to 100°/min, making it suitable for the specified 2°/min scan rate for analysis of powder samples. To evaluate the density of the glass system, Archimedes’ principle was employed. Toluene was selected as the immersion medium, and each sample was weighed in air and then in toluene. Each Measurement was repeated, and an average was taken for improved accuracy. The density ($\rho$, g/cm^3^) was determined using the following formula (1)^39^

1 $$\rho \left( {\frac{{\text{g}}}{{{\text{cm}}^{3} }}} \right) = \frac{{W_{a} }}{{W_{a} - W_{l} }} \times \rho_{l}$$

where $W_{a}$ and $W_{l}$ represents the respective weights of the samples in air and when suspended in toluene, where $\rho_{l} = 0.8669$ g/cm^3^.

Based on the experimentally determined density and molecular weight ($M_{w}$, g/mol), the molar volume ($V_{m}$) of the BBBNDy glass samples was evaluated using the Eq. (2)^40^:

2 $$V_{m} \left( {{\text{cm}}^{3} } \right) = \frac{{M_{w} }}{\rho }$$

Utilizing the measured density and molar volume, the average boron–boron separation ($D_{{\left\langle {B - B} \right\rangle }}$) and oxygen packing density (OPD) of the BBBNDy glasses were computed from relations (3) and (4)^41,42^

3 $$D_{{\left\langle {B - B} \right\rangle }} = \sqrt[3]{{\left( {\frac{{V_{m} }}{{2N_{A} \left( {1 - X_{B} } \right)}}} \right)}}$$

4 $$OPD = \frac{1000 \times n}{{V_{m} }}$$

where $N_{A}$ denotes Avogadro number, $X_{B}$ indicating the B_2_O_3_ molar fraction, and $n$ refers to the number of oxygen atoms per glass formulation, respectively.

The microstructure and surface morphological features and elemental analysis of the glass specimens were analyzed using a Carl Zeiss EVO 10 SEM equipped with secondary and backscatter electron detectors, offering a 3 nm resolution at 30 kV, 7 × 10^6^× magnification, and joystick-controlled stage movement (X: 80 mm, Y: 100 mm, Z: 35 mm). Energy-dispersive X-ray spectroscopy (EDX) was employed to analyze the mass percentages of the elements present, as per the X-ray interactions that reveal unique atomic structures. Fourier transform infrared (FTIR) measurements were carried out with a Bruker Alpha II spectrometer utilizing a diamond crystal attenuated reflectance (ATR) unit. The samples in the form of powder are directly used for analysis. Spectral acquisition covered the 400–4000 cm^−1^ region, employing a resolution of 4 cm^−1^, operating with OPUS version 7.8 software for data acquisition and analysis. Raman spectra were recorded using a Horiba Scientific Xplora plus confocal Raman spectrometer equipped with a 785 nm diode laser, across a 50–3000 cm^−1^ range. The spectral resolution was ≤ 1.4 cm^−1^/pixel equipped with a 2400 grooves/mm grating. All measurements are performed at room temperature.

The spectra of optical absorption about the investigated glasses in the 190–2500 nm range with a spectral resolution of 1 nm, utilizing a PerkinElmer Lambda 750S UV–VIS–NIR spectrophotometer. The absorption coefficient (*α*) was computed from absorbance (*a*) and sample thickness (*d*) as per Eq. (5)^43^,

5 $$\alpha = \frac{2.303 \times a}{d}$$

The direct and indirect optical energy band gap, $E_{opt}$ for the BBBNDy glasses were evaluated using the Mott and Davis relation^44^ outlined in Eq. (6), which links the absorption coefficient *α*(*ν*) and $E_{opt}$.

6 $$\left( {\alpha h\nu } \right)^{k} = D\left( {h\nu - E_{opt} } \right)$$

Here $h\nu$ represents the photon energy, $D$ is a proportionality constant, and $k$ defines the nature of the electronic transition, with values of 2 and 1/2 for direct and indirect allowed transitions, respectively.

Urbach energy ($E_{U}$) measures the degree of structural disorder or defects within amorphous materials. Transitions involving the localised tail states extending from the valence and conduction band edges into the forbidden energy region^45^, $E_{U}$ characterises this behaviour and is evaluated using relation (7), where G is a constant.

7 $$\alpha (\upnu ) = {\text{G}}\,e^{{\left( {h\nu /E_{U} } \right)}}$$

Steepness parameter ($S$), indicating electron–phonon induced broadening of the absorption edge^46^, is obtained from Eq. (8),

8 $$S = \frac{{k_{B} T}}{{E_{U} }}$$

where $k_{B}$ and T stands for the Boltzmann constant, and temperature in Kelvin, respectively.

Refractive index ($n$), an important optical parameter that is influenced by the internal electric field and the polarizability of ions in the material, was evaluated using the relation (9)^47^:

9 $$\frac{{n^{2} - 1}}{{n^{2} + 2}} = 1 - \sqrt {\frac{{E_{opt} }}{20}}$$

The metallization criterion ($M$), which predicts whether the material exhibits metallic or insulator behaviour, was evaluated by applying the following expression (10)^48^:

10 $$M = 1 - \frac{{R_{m} }}{{V_{m} }}$$

where $R_{m}$ and $V_{m}$ are the molar refraction and the molar volume, respectively.

Numerical aperture (*NA*), calculated using the relation (11)^49^

11 $$NA = n \times \left[ {2\delta } \right]^{{{\raise0.7ex\hbox{$1$} \!\mathord{\left/ {\vphantom {1 2}}\right.\kern-0pt} \!\lower0.7ex\hbox{$2$}}}}$$

$n$ stands for the refractive index, and $\delta$ signifies the fractional deviation in its value (0.01).

The surface reflection loss ($R_{L}$) from the glass was determined through the given formula (12)^50^:

12 $$R_{L} = \left( {\frac{n - 1}{{n + 2}}} \right)^{2}$$

An expression involving the refractive index ($n$) was employed to determine the optical transmission ($T$) (13)^51^:

13 $$T = \frac{2n}{{n^{2} + 1}}$$

Photoluminescence excitation and emission spectra were recorded using a JASCO FP-8500 spectrofluorometer, operating over the 200–800 nm wavelength range, equipped with a xenon flash lamp and a pulsed laser as excitation sources.

Glasses are irradiated with Gamma Chamber 5000 (BRIT, India) equipped with a Co-60 source, which ensures uniform gamma radiation exposure^52^. The samples underwent irradiation at a dose rate of 2.5 kGy/h for durations of 2, 4, and 6 h, corresponding to cumulative doses of 5, 10, and 15 kGy, respectively.

## Results and discussions

### Properties before irradiation

#### X-ray diffraction analysis (XRD)

**Fig. 2:**
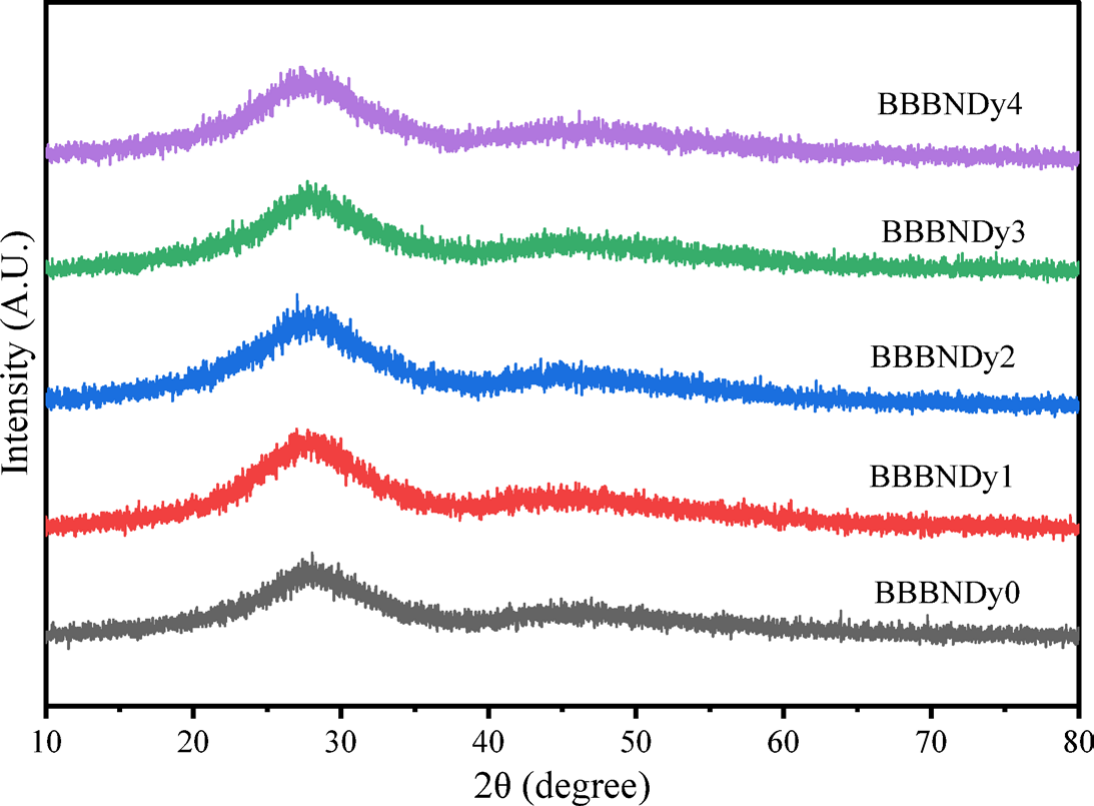
XRD spectra of BBBNDy samples.

The synthesized BBBNDy glasses exhibited an amorphous phase as evidenced by XRD patterns recorded between 10° and 80° range, as depicted in Fig. 2. The diffraction pattern for all BBBNDy glass samples exhibits broad and diffuse scattering at lower angles (~ 20° to ~ 35°). The absence of well-defined peaks indicates structural disorder in the glass network, thereby verifying the amorphous characteristics of the synthesized samples^8,40^.

#### Physical properties

##### Density (*ρ*) and molar volume (*V*_*m*_)

**Fig. 3:**
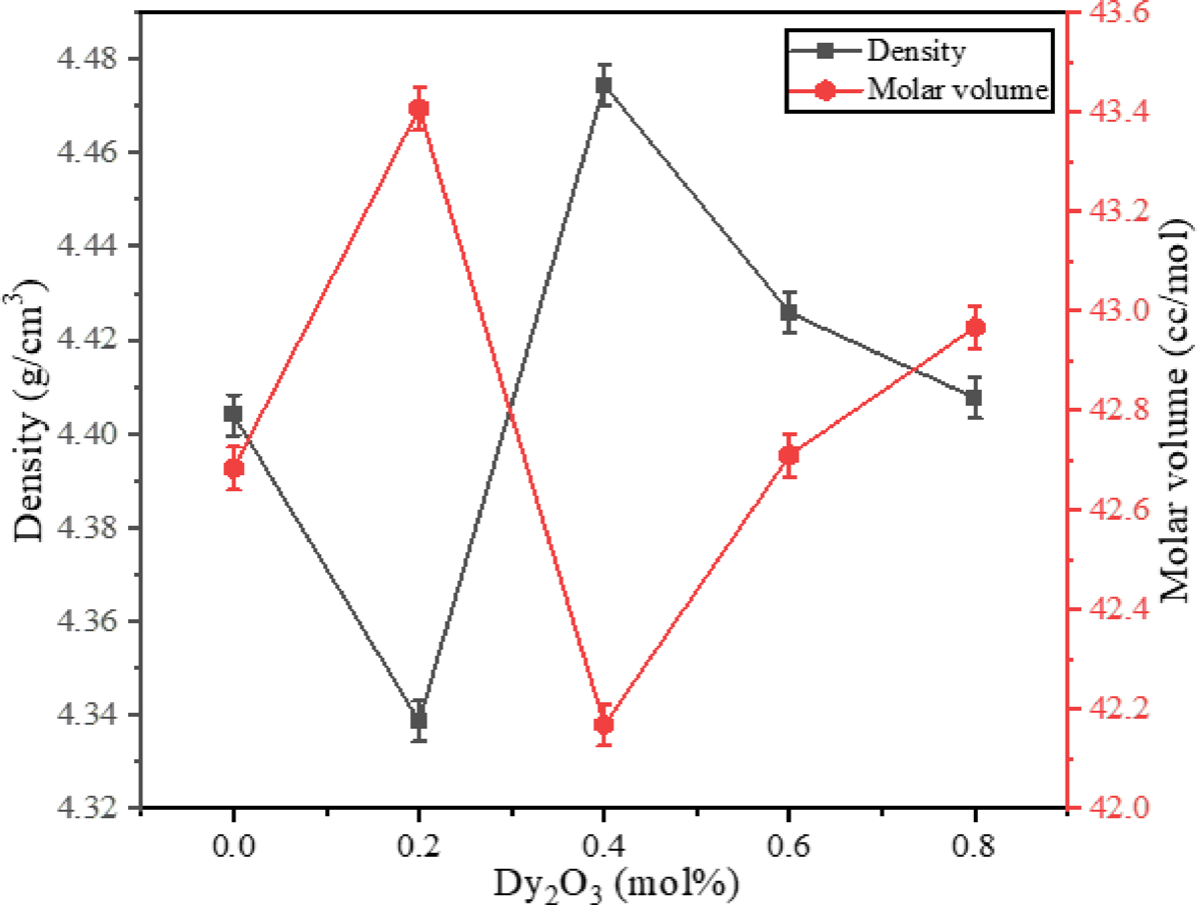
Influence of Dy_2_O_3_ concentration (mol%) on density and molar volume of BBBNDy glasses.

Table 2 and Fig. 3 reveal an inverse correlation between density and molar volume, resulting from the incorporation of Dy_2_O_3_ into the BBBNDy glasses, calculated using relations (1) and (2). The observed variations in the density of BBBNDy with increasing Dy_2_O_3_ content are primarily attributed to three factors: (1) The difference in molar mass between Dy_2_O_3_ and BaO, i.e., Dysprosium (162.5 u) is heavier than barium (137.3 u), generally increasing density. (2) The change in average molecular weight of the constituent oxides due to substitution of BaO with heavier Dy_2_O_3,_ and (3) The influence of Dy^3+^ ions on the structural arrangement of the glass matrix. However, trivalent dysprosium ions possess an ionic radius (1.03 Å) smaller compared to Ba^2+^ ions (1.35 Å), resulting in structural rearrangements. Dysprosium, owing to its compact ionic size and higher atomic weight relative to barium, influences overall compactness and mass distribution within the network. Additionally, Dy_2_O_3_ serves to modify the glass network, facilitating the formation of NBOs, thereby altering the local structural configuration and density, hence affecting the density behavior across the BBBNDy glasses. Such behavior is consistent with findings from other RE-doped glasses^40,43,53,54^.

**Table 2:** Physical properties of BBBNDy glasses.

Glass code	BBBNDy0	BBBNDy1	BBBNDy2	BBBNDy3	BBBNDy4
$\rho$, (g/cm^3^) (± 0.004)	4.404	4.339	4.474	4.426	4.408
$V_{m}$, (cc/mol)	42.684	43.406	42.169	42.711	42.967
$D_{{\left\langle {B - B} \right\rangle }}$_,_ (Å)	4.286	4.310	4.269	4.287	4.296
*OPD*, (g. atom/l)	56.227	55.384	57.104	56.473	56.223

The density of the BBBNDy glasses shows a minor change with increasing Dy_2_O_3_ concentration. It decreases from 4.404 g/cm^3^ for BBBNDy0 to 4.339 g/cm^3^ for BBBNDy1, then increases to a peak of 4.474 g/cm^3^ for BBBNDy2, followed by a slight decline at higher concentrations. As shown in Fig. 3, this behavior can be linked to structural changes across the glass network. At certain Dy_2_O_3_ concentrations, tighter atomic packing resulting from enhanced BO connectivity elevates the density values. At others, Dy_2_O_3_ disrupts the network, causing depolymerization, void formation, and generation of NBOs, causing density to decrease. The molar volume trends similarly reflect these structural changes as depicted in Fig. 3. Initially, Dy_2_O_3_ incorporation expands the structure by reducing the BO bonds, but at higher concentrations, the network stabilizes, reducing fluctuations in the molar volume as mentioned in Table 2.

##### Average boron–boron distance and oxygen packing density

**Fig. 4:**
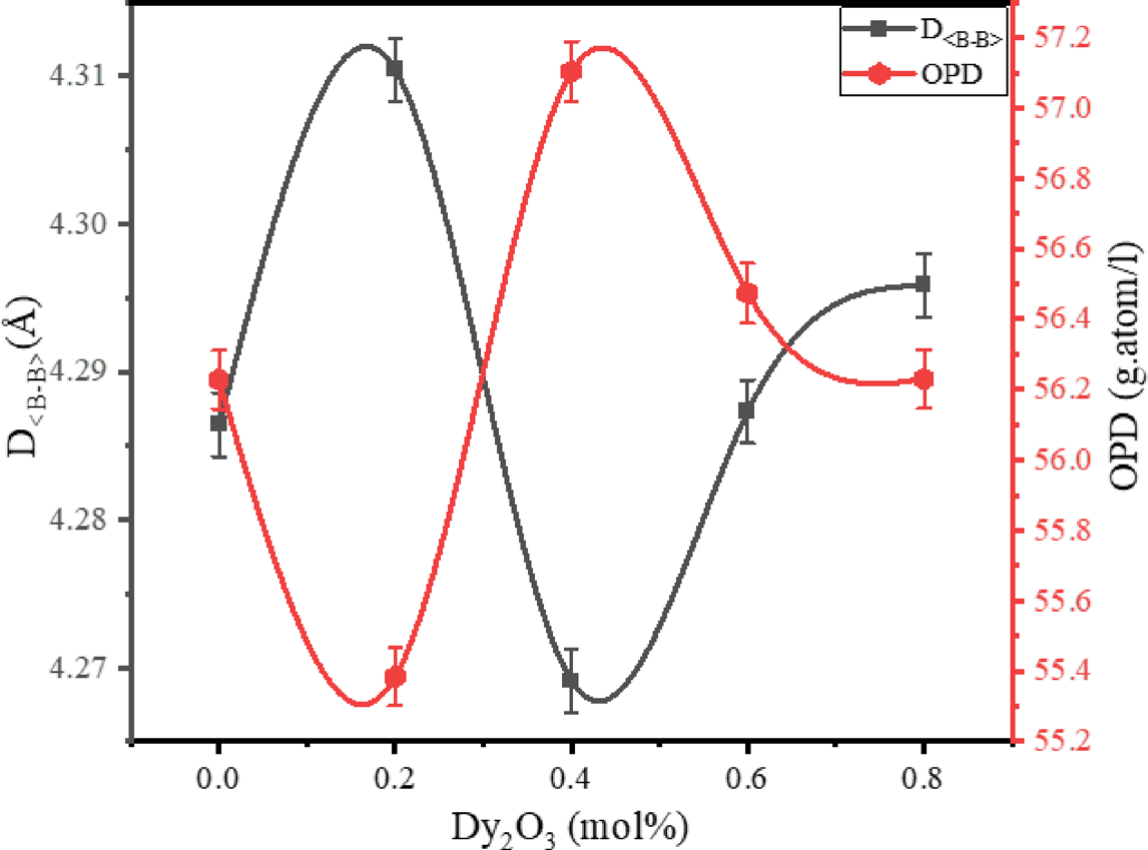
Influence of Dy_2_O_3_ concentration (mol%) on $D_{{\left\langle {B - B} \right\rangle }}$ and *OPD* of BBBNDy glasses.

Average boron-boron distance ($D_{{\left\langle {B - B} \right\rangle }}$) calculated using the empirical relation (3) signifies the separation connecting a pair of boron atoms in the network and indicates structural compactness. As shown in Table 2 and Fig. 4, $D_{{\left\langle {B - B} \right\rangle }}$ exhibits slight fluctuations with increasing Dy_2_O_3_ content. It initially increases from 4.286 Å for the undoped BBBNDy0 sample to a maximum of 4.310 Å at 0.2 mol% (BBBNDy1), then decreases and stabilizes around 4.296 Å (BBBNDy4). These subtle fluctuations suggest minor structural rearrangements within the borate glass network as modifier content increases.

Oxygen packing density (*OPD*), which measures how densely oxygen atoms are packed in the glass, was computed through the formula (4) and also shows compositional dependence as presented in Fig. 4, reflecting the tightness of the oxide network arrangement. It initially decreases, reaches a maximum value of 57.104 mol/L at 0.4 mol% of Dy_2_O_3_, and then stabilizes, as presented in Table 2. These variations reflect changes concerning the molar volume and packing density of the glass matrix, indicating alternating densification and modification induced by the incorporation of Dy_2_O_3_.

##### Scanning electron microscope (SEM) and energy dispersive spectroscopy (EDS)

When energetic electrons interact with atoms in borate glass samples, a range of signals is generated that reveal essential insights into the surface structure and elemental distribution of the material. SEM, in particular, plays a vital role in visualizing the surface morphology, while EDX provides a complementary compositional analysis. As each element has a distinct atomic structure, it results in a characteristic set of X-ray emission peaks.

**Fig. 5:**
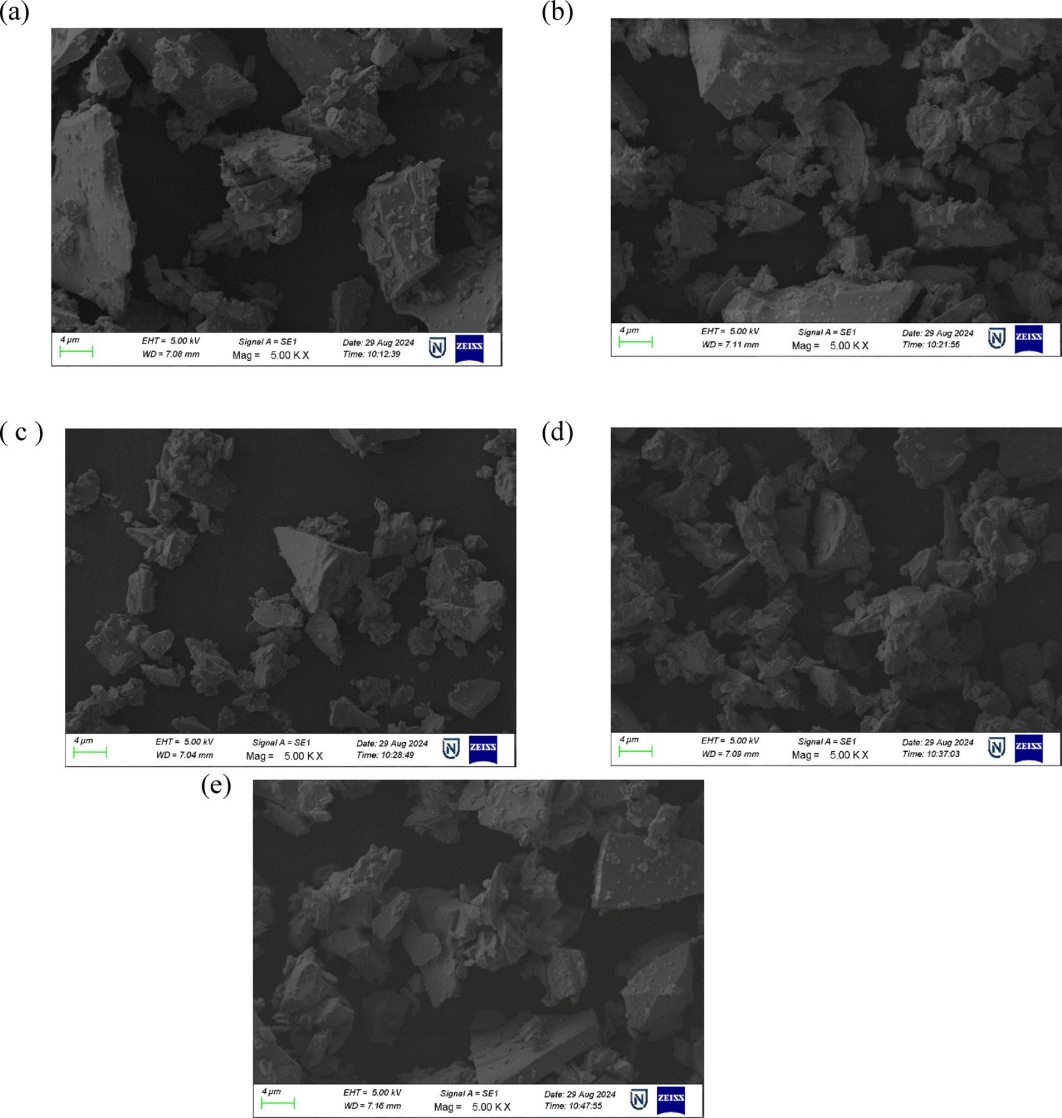
The SEM images of (**a**) BBBNDy0. (**b**) BBBNDy1. (**c**) BBBNDy2. (**d**) BBBNDy3. (**e**) BBBNDy4.

**Fig. 6:**
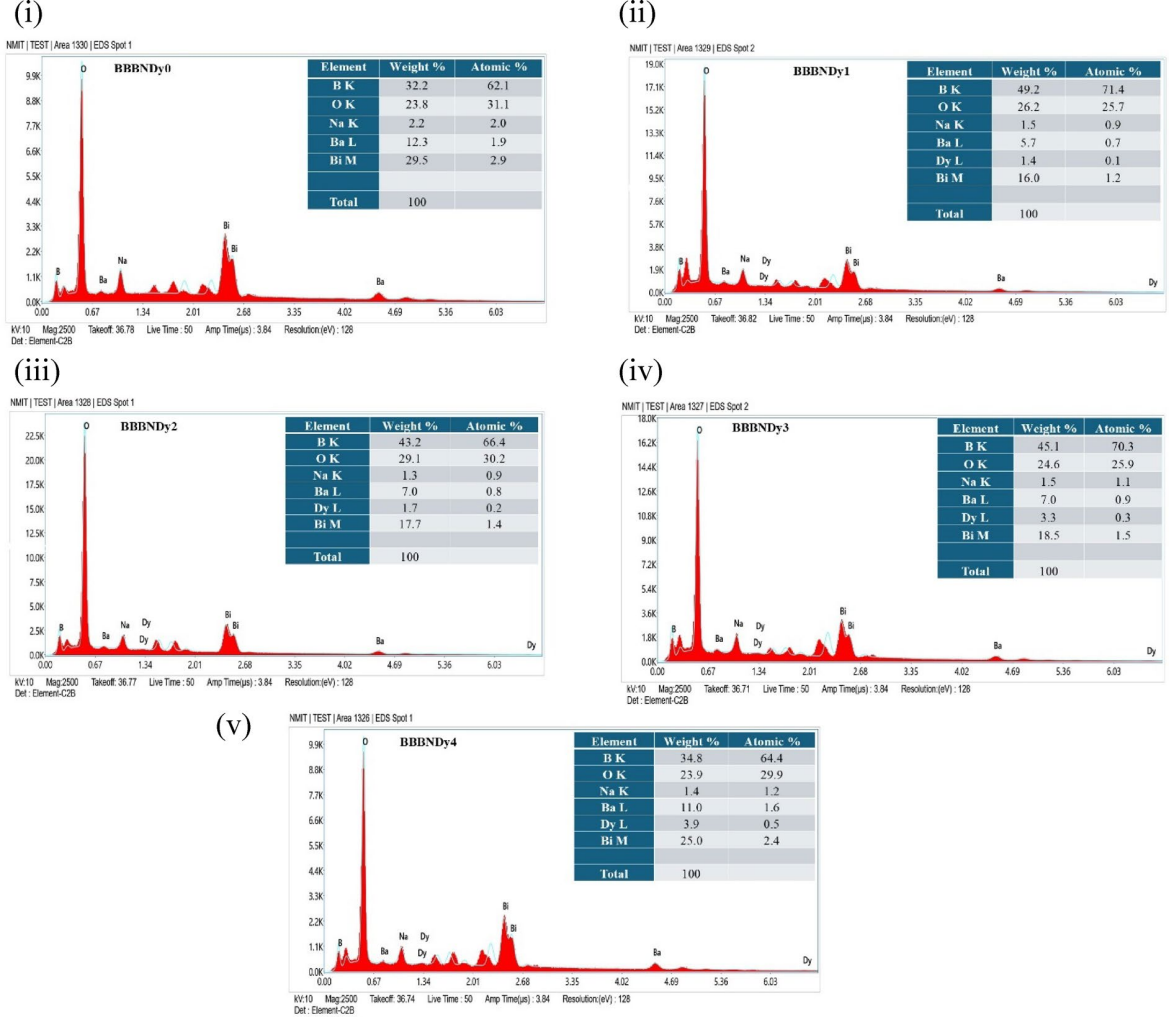
The EDS spectra of BBBNDy glasses (**i**–**v**) showing elemental peaks for B, Na, Ba, Bi, Dy, and O, confirming composition and Dy incorporation with increasing doping level.

SEM micrographs of the BBBNDy glasses revealed surfaces with dispersed grain-like features, with no evidence of long-range periodicity or crystalline phases^55^. At higher magnification of 5.00 KX, as shown in Fig. 5, irregular grain sizes, random distribution of surface particulates, and a continuous phase were observed in the range of a few hundred nanometers, which is characteristic of glass systems^56^. According to the EDS spectra in Fig. 6, the BBBNDy glasses contain key constituents including B, Ba, Na, Bi, Dy, and O and the corresponding EDS weight% values. A gradual increase in Dy peak intensity from BBBNDy1 to BBBNDy4 indicates successful Dy_2_O_3_ incorporation. The Dy content increased from 0.8 wt% (BBBNDy1) to 3.9 wt% (BBBNDy4), in agreement with the nominal doping levels. Minor variations in peak intensity indicate slight elemental inhomogeneity, a characteristic of amorphous systems. This non-uniform distribution may influence the structural arrangement of glass and related properties, as elaborated in subsequent sections. Lack of extraneous peaks confirms the chemical purity within the samples and indicates the absence of elements from porcelain crucibles^43,57,58^.

##### FTIR spectroscopic analysis

**Fig. 7:**
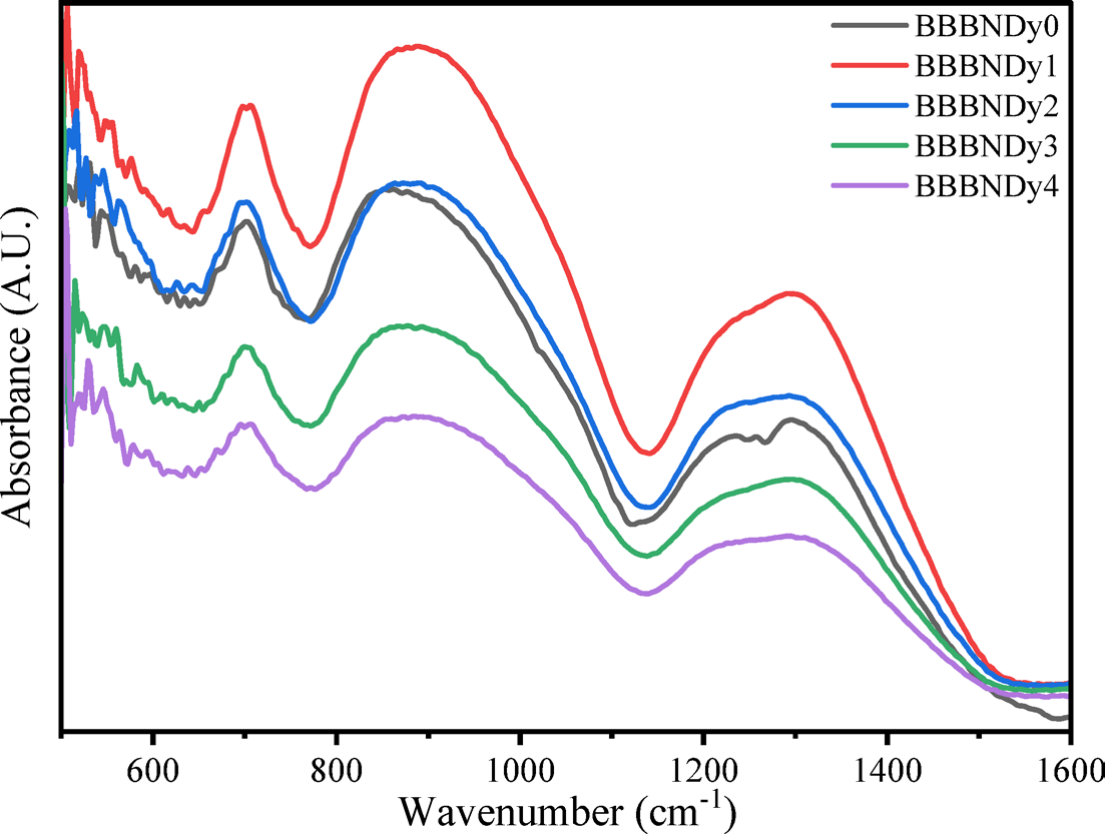
FTIR spectra of BBBNDy glasses.

**Fig. 8:**
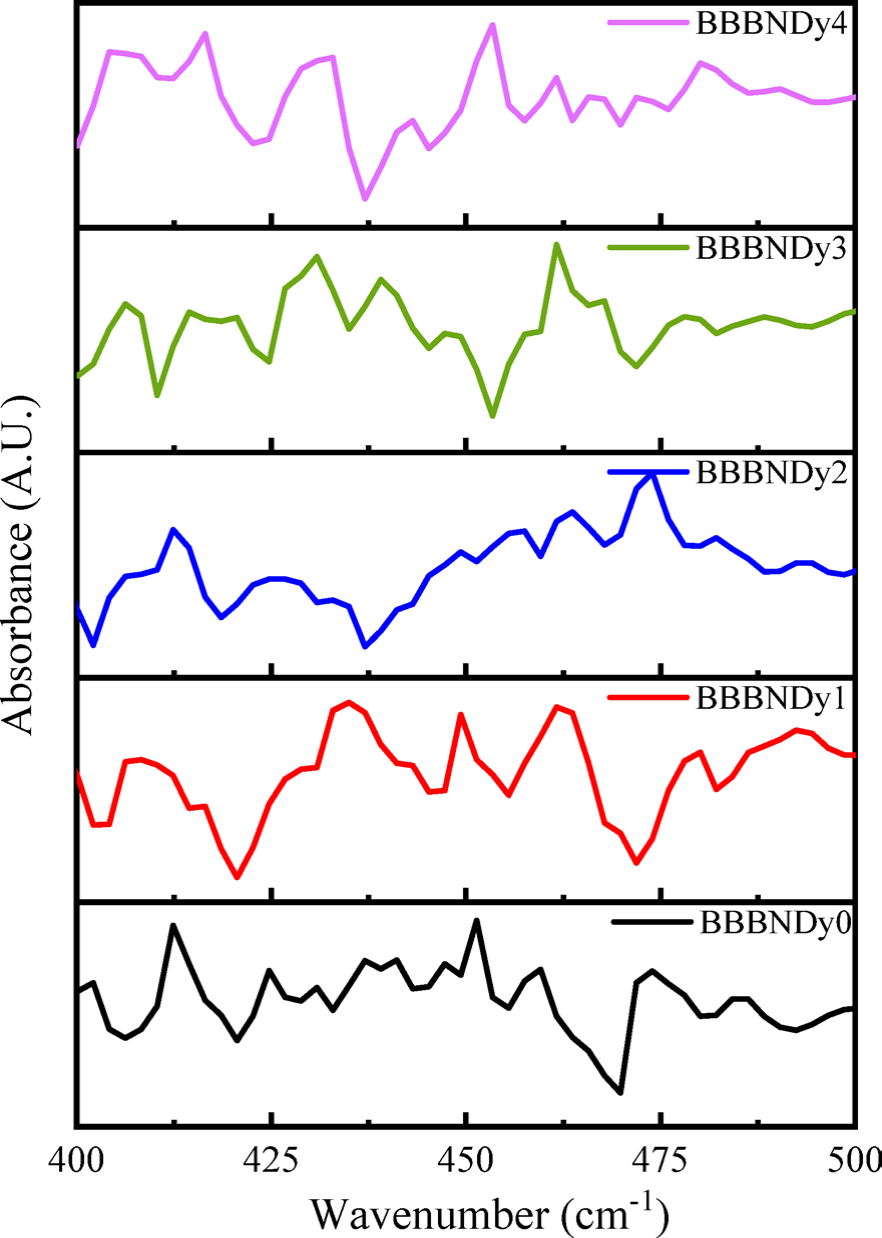
Cationic vibrations of FTIR spectra.

Fourier Transform Infrared (FTIR) spectroscopy is a widely adopted and insightful method in materials research for analyzing structural modifications, enabling the differentiation of various functional groups present in glass materials. This technique relies on the principle that each molecule possesses distinct vibrational modes, which serve as a unique fingerprint for its identification. The glass networks’ vibrational modes are independent of those corresponding to other functional groups in other regions^43^. The region in the mid-infrared, i.e., (400–1600 cm^−1^), is of particular interest, as the vibrational modes in bismuth-borate glasses are primarily active within this range^59^. Figure 7 presents the FTIR spectra of BBBNDy glasses, while Fig. 8 illustrates their cation-related vibrational modes. Several studies^60,61^ report that Bi_2_O_3_ in B_2_O_3_ can participate in the glass structure in three distinct ways: (1) Oxygen donation to borate structure promotes the development of four-coordinated boron (BO_4_) units. (2) By forming BiO_3_ pyramidal structures, characterized by C_3V_ symmetry, and (3) Through the addition of NBO atoms, the network connectivity is reduced^60,61^.

The absorption band around ~ 450 cm^−1^ is associated with the vibration of metal cation Na^2+^, Ca^2+^ ions^62,63^. The absorption bands below ~ 650 cm^−1^ signify vibrations of the Bi–O bond, originating from both BiO_6_ octahedral and BiO_3_ pyramidal units^64^. BiO_3_ structural units are characterized by four primary vibrational modes: a symmetric vibration around ~ 840 cm^−1^, a two-fold degenerate stretching vibration within the ~ 540 to ~ 620 cm^−1^ range, a symmetrical bending mode near 470 cm^−1^, and a bending mode exhibiting double degeneracy approximately at ~ 350 cm^−1^^65–67^. In the present investigation, the manifestation of these modes may vary, with only certain bands being detectable based on the local structural environment and specific glass composition.

**Fig. 9:**
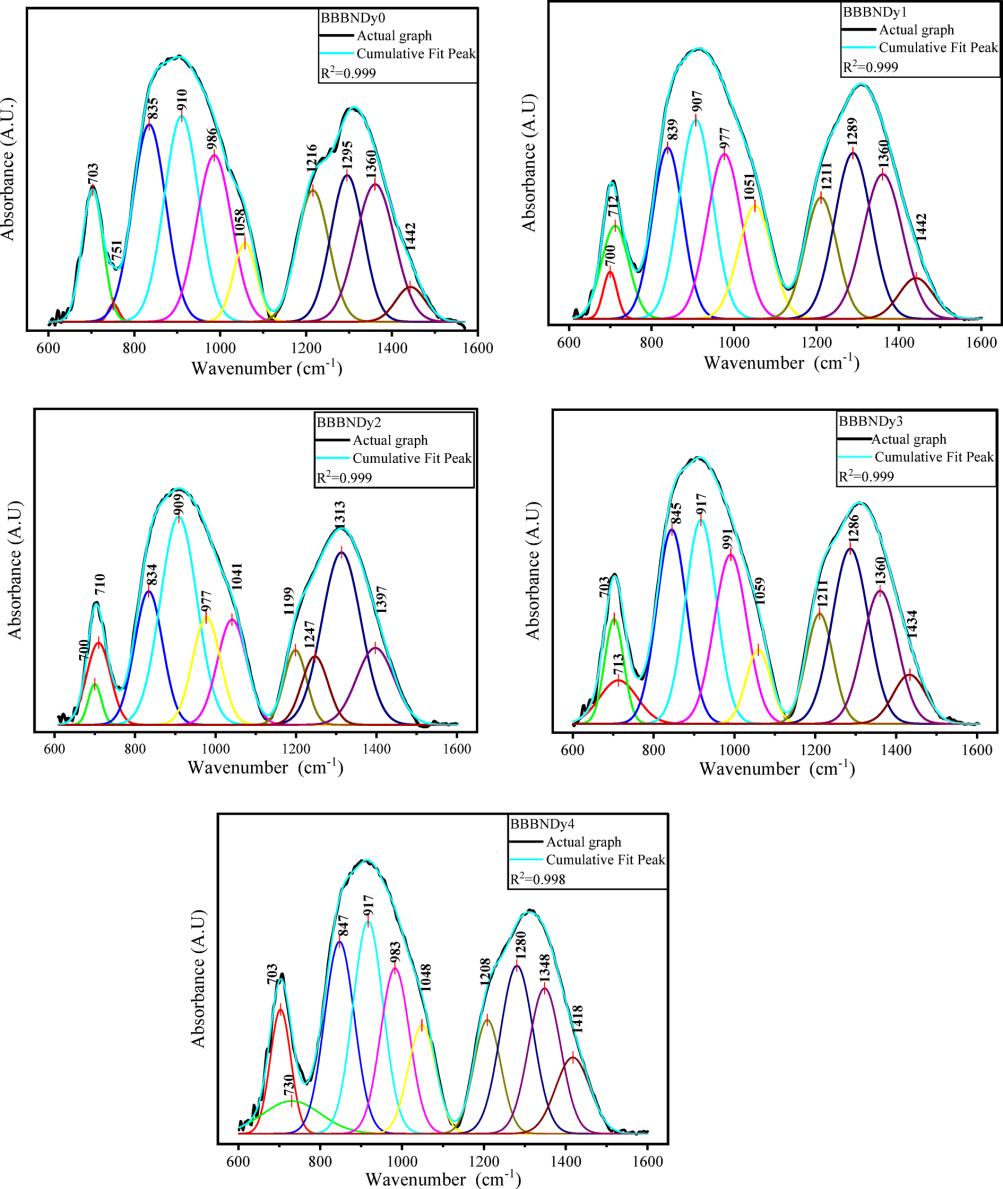
Deconvoluted FTIR spectra of BBBNDy glasses.

To resolve overlapping peaks and extract detailed structural information, the FTIR spectra were deconvoluted using Gaussian peak fitting. As shown in Fig. 9, this analysis resolved vibrational modes in the 800–1100 cm^−1^ and 1200–1600 cm^−1^ regions, confirming the presence of BO_4_, BO_3,_ and Bi–O structures. Deconvoluted results reinforce the assignments made from the raw spectra and provide deeper insight into the glass matrix. The absorption band observed ~ 699 cm^−1^ to ~ 702 cm^−1^ is attributed to the symmetric Bi–O stretching modes characteristic of BiO_3_ pyramidal units; thereafter, bending vibrations of O_3_B–O–BO_3_ may contribute to overlapping spectral features^68–70^. The region between ~ 709 and 751 cm^−1^ is attributed to stretching vibrations of B–O bonds in BO_4_ tetrahedral configurations^71^. The region between ~ 833 and 1059 cm^−1^ is attributed to BO_4_ stretching vibrations from B–O linkages. These units are likely connected via bismuth cations, implying the existence of B–O–Bi linkages incorporated into the glass network^64,72^.

The absorption band within ~ 1198 cm^−1^ to 1295 cm^−1^ corresponds to asymmetric B–O stretching vibrations in BO_3_ units, commonly linked to ortho and pyro-borate species^71^. ~ 1312 cm^−1^ to 1361 cm^−1^ ascribed to the asymmetric B–O stretching vibrations in BO_3_ groups within boroxyl rings, indicating the presence of three-membered borate ring structures^71^. ~ 1397–1443 cm^−1^, region corresponds to vibrational modes involving Bi–O bonds in BiO_6_ and BiO_3_ structural units, as well as stretching B–O vibrations in BO_3_ groups originating through various borate species^71,73^. The assigned vibrational bands and their peak positions are listed in Table 3. The band positions slightly shift with the dopant concentrations, showing the structural rearrangements.

**Table 3:** Vibrational peaks identified in the BBBNDy glasses with assignments.

Wavenumber (cm^−1^)	Assignments	References
~ 450	Vibrations of Na^2+^, Ca^2+^ metal cations	^62,63^
< 650	Vibrations of the Bi–O bond from BiO_6_ octahedral and BiO_3_ pyramidal structures	^64^
~ 699 to ~ 702	Symmetric Bi–O stretching modes associated with BiO_3_ pyramidal units, possibly superimposed with bending O_3_B–O–BO_3_ modes	^68–70^
~ 709–751	Stretching B–O vibrations in borate tetrahedral units	^71^
~ 833–1059	stretching vibrations of B–O bonds in BO_4_ tetrahedral units, with linkages of B–O–Bi structural units	^64,72^
~ 1198 to 1295	Asymmetric B–O stretching vibrations in BO_3_ units (ortho and pyro-borate configurations	^71^
~ 1312 to 1361	Asymmetric B–O stretching vibrations in BO_3_ unit	^71^
~ 1397 to 1443	Vibrations of B–O stretching in BO_3_ groups and Bi–O modes in BiO_6_ and BiO_3_ structures	^71,73^

As reported by Pascuta et al. and other studies^74–76^ and illustrated in Fig. 7, the IR spectra reveal that the introduction of RE ions induces minimal changes in the absorption features of the glasses owing to the rigidity of the RE-doped bismuth borate matrix.

##### Raman spectral analysis

**Fig. 10:**
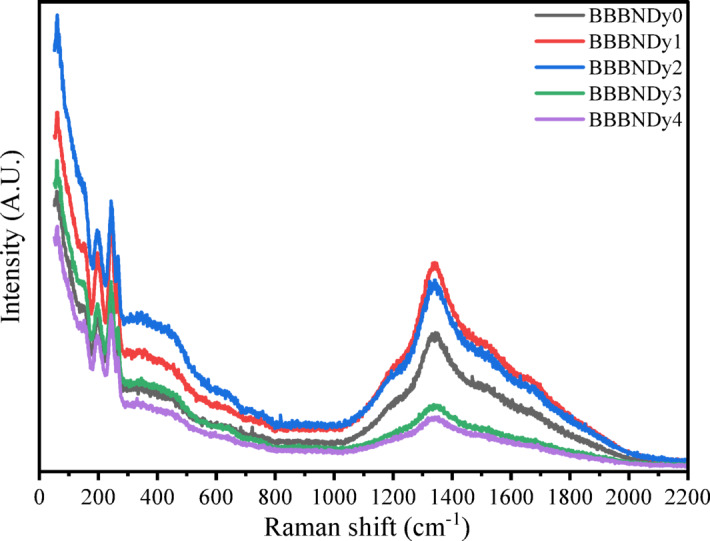
Raman spectral analysis for BBBNDy glasses.

Raman spectroscopy was utilized to analyze the vibrational modes and structural network of the BBBNDy glasses. Spectra in Fig. 10 were recorded in the 50–2200 cm^−1^ range, where key vibrational features of borate and bismuth units are typically active. The broad and diffuse features in the spectra indicate the non-crystalline structure of the glasses. The corresponding Raman peak positions and their vibrational assignments are summarized in Table 4.

**Table 4:** Raman peak positions and associated band assignments of BBBNDy glasses.

Raman shift (cm^−1^)	Peak attributions	References
~ 65–98	Modes of vibration in Bi^3+^ ions exhibiting E_g_ symmetry	^63,71^
~ 154–199	Bi^3+^ ion vibrations with BiO_3_ and BiO_6_ structural units, as well as with Bi–O stretching modes	^77,78^
~ 244–266	Bi–O–Bi bond vibrations arising from BiO_6_ octahedral and BiO_3_ pyramidal structural units	^79,80^
~ 339–450	Bi–O–Bi bond linkages with BiO_6_ structural units, overlapping with the vibrational modes of [BO_3_] and [BO_4_] structural motifs	^64,81^
~ 628	B–O vibrational modes with [BO_4_] tetrahedral units and B–O–B linkages, in different borate species	^71^
~ 754	Symmetrical B–O stretching modes in BO_4_ units, particularly linked to orthoborate groups, are influenced by pyramidal modes of Bi_2_O_3_	^71,82^
~ 1195	Asymmetric B–O stretching modes in terminal [BO_3_] motifs, linked with pyroborate structural units	^83^
~ 1337–1387	Stretching B–O vibrations with tetrahedral [BO_4_] units	^84^
~ 1657	Stretching B–O vibrations associated with pyro-borate structural units	^85^

Low frequency band at ~ 65–98 cm^−1^ corresponds to vibrational modes of Bi^3+^ ions exhibiting E_g_ symmetry, typically associated with doubly degenerate bending modes within BiO_6_ octahedral units^63,71^. The range ~ 154–199 cm^−1^ is related to vibrational behavior of the Bi^3+^ ion coordinated in BiO_3_ pyramids and BiO_6_ octahedra structures, along with Bi–O stretching modes with these structural configurations in the glass matrix^77,78^. Bands observed at ~ 244–266 cm^−1^ correspond to Bi–O–Bi vibrations arising from both BiO_6_ octahedra and BiO_3_ pyramid structural units in the glass network^79,80^. Region ~ 339–450 cm^−1^ bands, attributed to linkages of Bi–O–Bi associated with BiO_6_ octahedra configurations, overlapping with the vibrational features of [BO_3_] and [BO_4_] coordination environments in the glass^64,81^. ~ 628 cm^−1^ peak is assigned to B–O vibrations due to stretching with [BO_4_] tetrahedral units and linkages of B–O–B, commonly observed due to meta and tetraborate groups^71^. The band ~ 754 cm^−1^ arises from the symmetric vibrational stretching of B–O bonds in BO_4_ tetrahedra, particularly associated with orthoborate groups, and is likely influenced by pyramidal modes of Bi_2_O_3_^71,82^.

In general peak near 800 cm^−1^ evidences the existence of boroxyl units in the borate glass. However, the spectra of the investigated glasses exhibit no distinct features in this region, indicating the lack of boroxyl rings in the structure of the studied glasses. The peak observed at ~ 1195 cm^−1^ is assigned to the Asymmetric stretching of terminal B–O bonds in [BO_3_] units, specifically associated with the pyroborate structural units^83^. In the region ~ 1337–1387 cm^−1^_,_ bands correspond to B–O stretching vibrations with tetrahedral [BO_4_] units, indicating the presence of well-defined tetrahedral borate configurations^84^. The peak at ~ 1657 cm^−1^ corresponds to the stretching vibrations of terminal B–O bonds associated with pyro-borate structural units, indicating their presence within the glass network^85^. Nonetheless, the Raman spectral features of BBBNDy glasses with varying Dy_2_O_3_ concentrations remain largely unchanged, indicating that the addition of Dy_2_O_3_ does not significantly alter the glass structure, likely due to the relatively low concentration compared to glass formers.

#### Optical properties

**Fig. 11:**
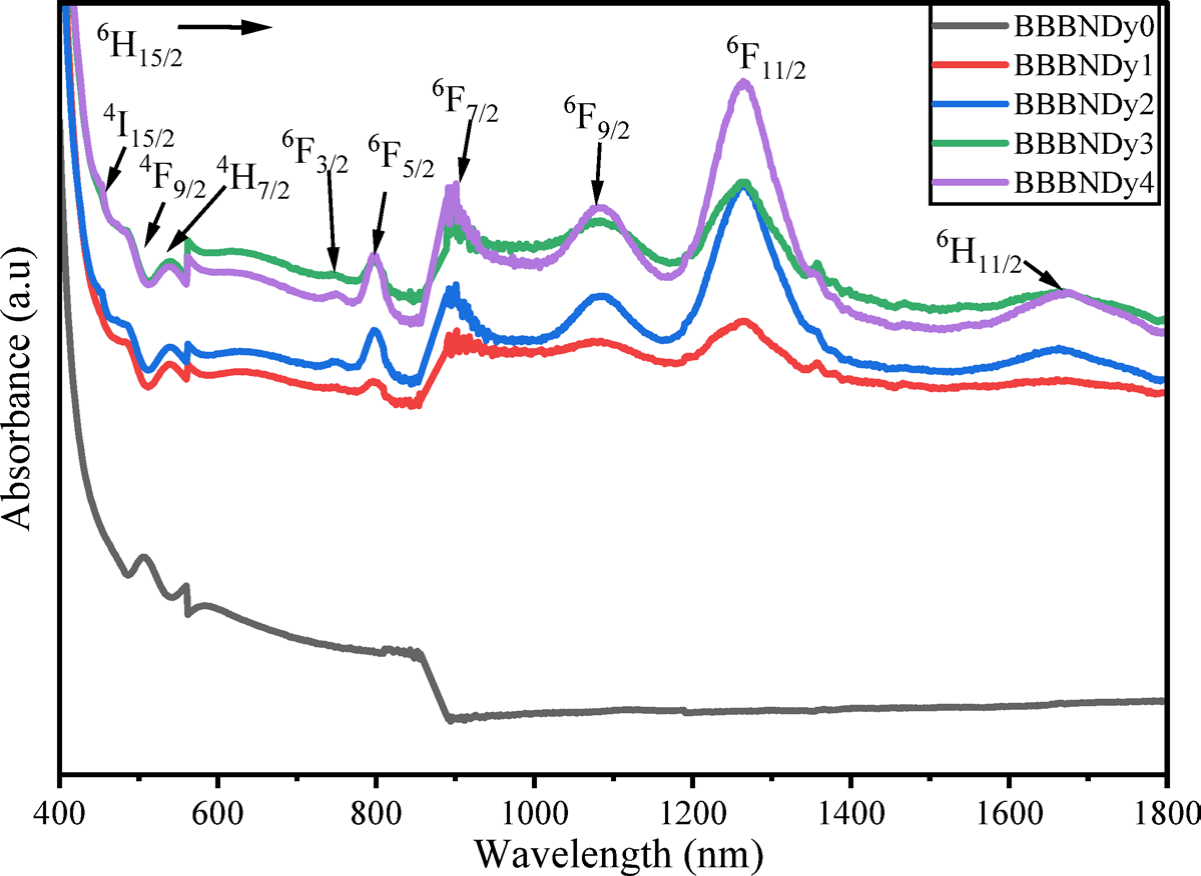
Absorption spectra of BBBNDy glasses with increasing Dy_2_O_3_ content.

The absorption spectra of Dy_2_O_3_-doped glass samples recorded in the spectral range of 400–1800 nm are shown in Fig. 11. A series of absorption bands are observed, corresponding to the characteristic 4*f*–4*f* transition of Dy^3+^ ions starting from the ^6^H_15/2_ ground state to various higher energy levels. These transitions include: ^4^I_15/2_, ^4^F_9/2_, ^4^H_7/2_, ^6^F_3/2_, ^6^F_5/2_, ^6^F_7/2_, ^6^F_9/2_, ^6^F_11/2_, ^6^H_11/2_ at wavelengths 447 nm, 473 nm, 539 nm, 751 nm, 799 nm, 889 nm, 1086 nm, 1263 nm, and 1672 nm, respectively. These transitions are consistent with earlier reports^86–88^.

The spectra exhibit higher intensity and broader peaks in the NIR region compared to the UV–visible region. Such a response arises from the hypersensitive nature of the ^6^H_15/2_ → ^6^F_11/2_ transition near 1263 nm, which shows pronounced variation in intensity with the surrounding environment of Dy^3+^ ions. This transition follows the electric dipole selection rules $\left| {\Delta S} \right|$= 0, $\left| {\Delta L} \right| \le 2,$ and $\left| {\Delta J} \right| \le 2$, making Dy^3+^ ions hypersensitive to changes in the local glass structure^89,90^. Increasing Dy_2_O_3_ content from 0 to 0.8 mol%, a steady rise in absorption peak intensities is observed, indicating successful incorporation of Dy^3+^ ions into the glass matrix.

##### Optical energy band gaps (***E***_***opt***_), Urbach energy (***E***_***U***_) and steepness parameter (***S***)

The optical energy band gap ($E_{opt}$) plays a critical role in understanding the electronic transitions and bonding nature in oxide glasses. It denotes the minimum energy needed to transfer an electron across the band gap. In amorphous systems like Dy_2_O_3_-doped BBBNDy glasses, the evaluation of $E_{opt}$ helps reveal variations in structural reorganisation and chemical bonds of the network owing to the non-crystalline nature of the material.

**Fig. 12:**
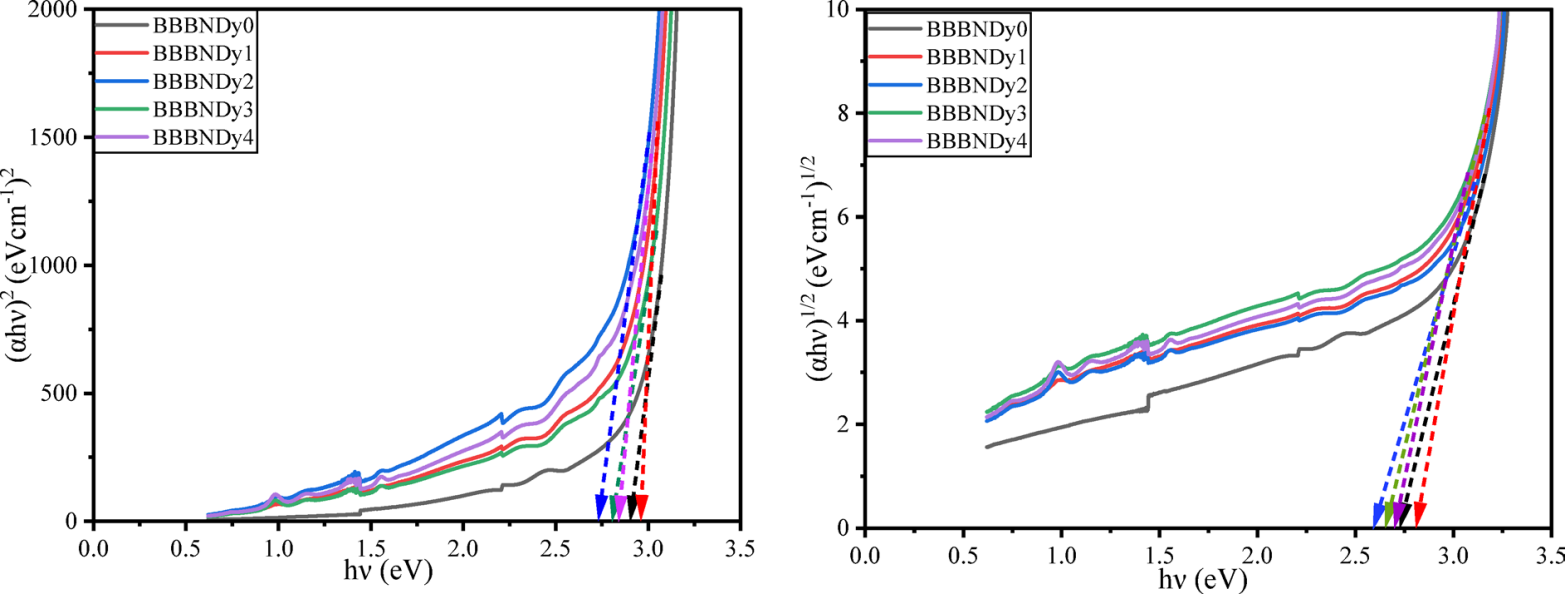
Tauc plots for BBBNDy glasses to determine optical energy band gaps.

To determine $E_{opt}$, UV–VIS–NIR spectral analysis was recorded for the as-prepared glasses. The absorption coefficient (α) was estimated based on the absorbance (a) and thickness (d) of the sample using the relation (6). Tauc plots were constructed by plotting $\left( {\alpha h\nu } \right)^{k}$ versus $h\nu$ to estimate the optical energy band gap, by linearly extrapolating the curves to the X-axis as depicted in Fig. 12.

For the BBBNDy glass system, the direct energy band gap ($$E_{opt} )$$ values were found between 2.729 and 2.957 eV. Similarly, the indirect energy band gap ($$E_{opt} )$$ values were found to be 2.589 to 2.807 eV. The non-linear variations in both direct and indirect energy band gaps were observed, which aligns well with the FTIR results. In glasses, the optical band gap is primarily influenced by two key factors: the distribution of anions and cations involved in the network, and the proportion of NBOs present in the structure. The band gap of the glass decreases with increasing average atomic number, and the presence of NBOs, as electrons associated with NBOs require less energy to make a transition to the conduction band compared to those from BOs. Variations in band gap can also arise from changes in symmetry and local field strength due to interactions between the rare earth (RE) ions and the glass matrix^91,92^. The observed increase in the band gap at specific Dy_2_O_3_ concentrations is attributed to the formation of BO units, which enhance the network compactness and lead to band widening^91,92^.

Urbach energy ($E_{U}$) was determined from the linear portion of the ln (α) vs. hν plot, followed by taking the inverse of the slope. Calculated E_U_ values are tabulated in Table 5, ranging from 0.175 to 0.227 eV. A Lower Urbach energy suggests a reduced density of localized states in the band tails, and structural stability^93,94^, indicating that Dy_2_O_3_ doping introduces more defects into the glass matrix, thereby affecting its optical properties.

**Table 5:** Optical properties of BBBNDy glasses.

Sample code	BBBNDy0	BBBNDy1	BBBNDy2	BBBNDy3	BBBNDy4
Direct band gap (eV) (± 0.001)	2.895	2.957	2.729	2.802	2.833
Indirect band gap (eV)	2.735	2.807	2.589	2.662	2.704
Urbach energy, $E_{U}$ (eV)	0.175	0.205	0.227	0.183	0.214
Steepness parameter, $S$	0.148	0.126	0.114	0.141	0.121
Refractive index, $n$	2.426	2.409	2.474	2.453	2.444
Metallization criteria, *M*	0.380	0.385	0.369	0.374	0.376
Numerical aperture, *NA*	0.343	0.341	0.349	0.347	0.346
Reflection loss, $R_{L}$	0.173	0.171	0.180	0.177	0.176
Optical transmission, *T*	0.7047	0.7083	0.6949	0.6992	0.7011

Steepness parameter ($S$) computed from relation (8) and tabulated in Table 5, demonstrated an inverse proportionality to the E_U_ values, ranging from 0.114 to 0.148. BBBNDy glass samples increased disordering results in the development of defect-induced states in the band gap^41^ and hence a decrease in the optical band gap.

##### Refractive index (*n*), metallization criteria (*M*), and numerical aperture (*NA*)

The refractive index of a material shows a direct correlation with its density; an increase in density generally results in a higher refractive index. Glass with a higher refractive index indicates that light travels more slowly through the medium, signifying greater optical density. The refractive index calculated using Eq. (9) was found to vary between 2.409 and 2.474 with the inclusion of Dy_2_O_3_ content in BBBNDy glasses, and it is highest for BBBNDy2 glass with a value of 2.474. Here, the increase in the value of *n* is attributed to structural compactness and denser packing of atoms or ions within the network, and vice versa. As glass becomes denser, its ability to polarize in response to an electromagnetic field also increases, leading to an enhanced optical response. The values of *n* in BBBNDy glasses are calculated using the direct energy band gap.

The insulating characteristics of the synthesized BBBNDy glass samples were evaluated using the metallization criteria, by Eq. (10). The calculated *M* values for Dy_2_O_3_-doped BBBNDy glasses were found between 0.369 and 0.385, well below the critical threshold of 1(one)^42^. This confirms the insulating behavior of the glasses. The lower values of M with Dy_2_O_3_ addition suggest a more pronounced non-metallic nature, likely due to enhanced structural compactness in the network.

The numerical aperture (*NA*), which measures the light-gathering capability of the material and is also linked to the refractive index (*n*) via Eq. (11), varied between 0.341 and 0.345. The *NA* follows a similar trend with refractive index, increasing with Dy_2_O_3_ content up to a certain level, then stabilizing at higher concentrations. This trend signifies good light collection efficiency, which is advantageous for optical applications^95^. The refractive index, metallization criteria, and the numerical aperture values of the BBBNDy glass system with increasing Dy_2_O_3_ content are tabulated in Table 5.

##### Reflection loss (***R***_***L***_) and optical transmission (***T***)

The reflection loss ($R_{L}$), which quantifies the amount of light reflected from the material’s surface influenced by its refractive index^48^, is described by the Eq. (12). The $R_{L}$ values in the BBBNDy glass system vary slightly from 0.171 to 0.180, which correlates with the refractive index behavior. The increase in refractive index with Dy_2_O_3_ content results in a marginal rise in reflection loss, peaking for BBBNDy2 glass at 0.180 before stabilizing.

The optical transmission (*T*) values varied from 0.695 to 0.708, calculated from the relation (13). A lower $R_{L}$ or high *T* is preferred for optimal optical transmission, and the observed values suggest satisfactory optical clarity in the glasses. $R_{L}$ and *T* findings are tabulated in Table 5.

##### Photoluminescence (PL) analysis

**Fig. 13:**
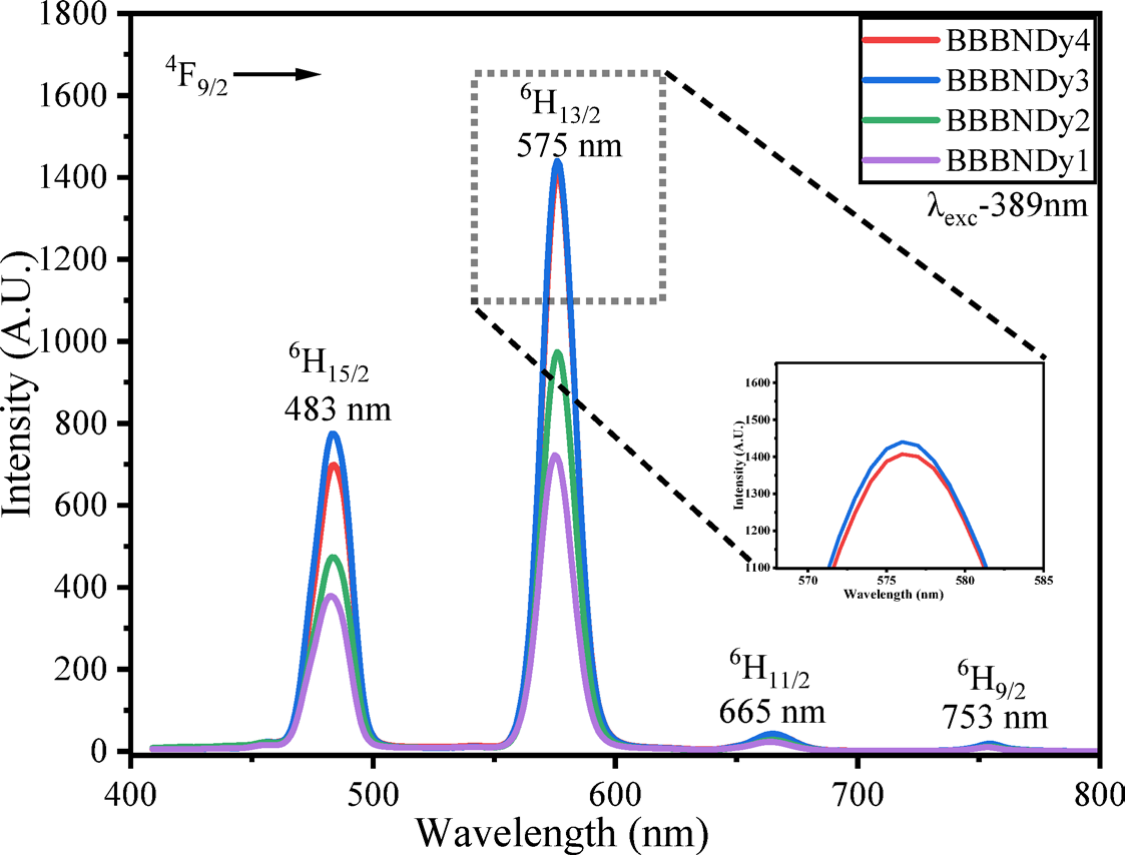
Emission spectra for the BBBNDy glass series.

The emission profiles obtained from PL analysis of Dy_2_O_3_-doped BBBNDy glasses were examined under an excitation wavelength of 389 nm and are depicted in Fig. 13. The spectra reveal four distinct emission bands in the range of 450–750 nm, corresponding to the characteristic 4*f*–4*f* transitions of Dy^3+^ ions. The key emission peaks observed at approximately 483 nm (blue), 575 nm (yellow), 665 nm (red), and 753 nm are associated with the electronic transitions ^4^F_9/2_ → ^6^H_15/2_; ^6^H_13/2_; ^6^H_11/2_ and ^6^H_9/2_, respectively^96,97^.

**Fig. 14:**
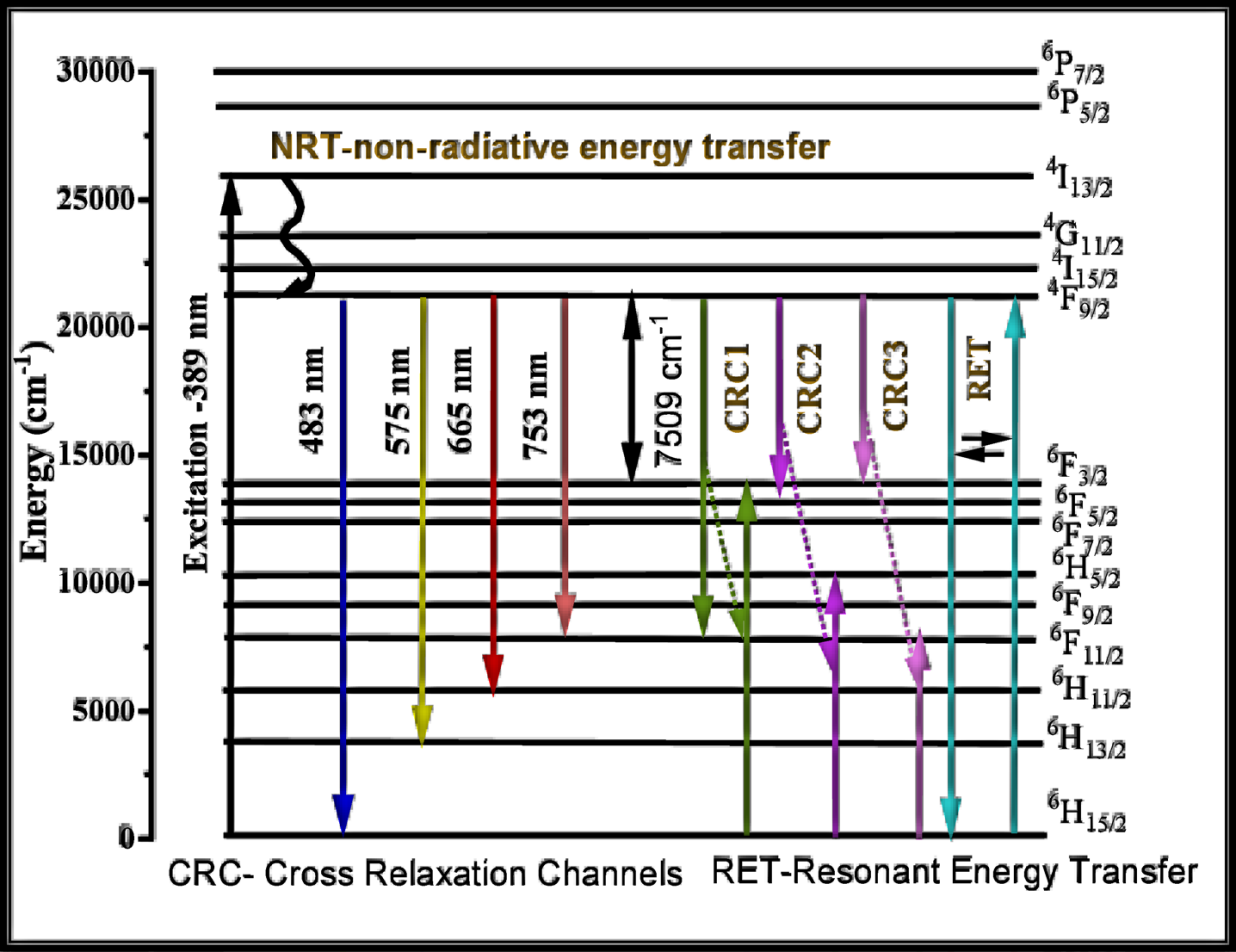
Energy level diagram of Dy^3+^ ions in BBBNDy glasses showing main radiative emissions(blue, yellow, red) and possible non-radiative relaxation pathways.

Among these, the transition ^4^F_9/2_ → ^6^H_13/2_, exhibiting electric dipole behavior and following the selection rules ∆J = 0 or ± 2, is hypersensitive, with the highest intensity. This hypersensitivity renders it highly sensitive to the asymmetry and the surrounding environment around the Dy^3+^ site. Conversely, a magnetic dipole transition with low intensity ^4^F_9/2_ → ^6^H_15/2_, follows the selection rules ∆J = 0 or ± 1, and is minimally influenced by such factors^98,99^. Figure 13 demonstrates that emission intensity increased with Dy_2_O_3_ content up to 0.6 mol%; however, a further increase results in a decline, which can be ascribed to concentration quenching effects, primarily resulting from RET (resonance energy transfer) and CRC (cross relaxation channels) mechanisms among Dy^3+^ ions. From Fig. 14, the observed energy transfer pathways include the following:

(a) ^4^F_9/2_:^6^H_15/2_ → ^6^H_15/2_:^4^F_9/2_, (b) ^4^F_9/2_:^6^H_15/2_ → ^6^F_3/2_:^6^F_11/2_, (c) ^4^F_9/2_:^6^H_15/2_ → ^6^F_5/2_:^6^F_9/2_, and

(d) ^4^F_9/2_:^6^H_15/2_ → ^6^F_11/2_:^6^F_3/2_^96^.

##### Yellow to blue (Y/B) intensity ratio and CCT diagram

The Y/B intensity ratio is a useful indicator of the asymmetry in the Dy^3+^ ion environment and is calculated for the BBBNDy glasses, as presented in Table 6. A higher Y/B ratio suggests enhanced asymmetry around Dy^3+^ ionic sites, suggesting enhanced polarizability, higher covalency, and the dominance of the transition of electric dipole in the glass network^100,101^. The observed trend in the values of the Y/B ratio shows that the maximum asymmetry is achieved at an intermediate doping level (BBBNDy3). Beyond this point (BBBNDy4), a decrease in the Y/B ratio suggests the onset of concentration quenching, which diminishes the effective asymmetry contribution.

**Table 6:** Chromaticity coordinates (x, y), emission intensity ratios (Y/B), and color correlated temperature (CCT) for BBBNDy glass samples.

Sample code	x	y	Y/B ratio	CCT
BBBNDy1	0.396	0.426	1.89	3948
BBBNDy2	0.390	0.422	2.05	4040
BBBNDy3	0.392	0.421	2.05	3995
BBBNDy4	0.383	0.420	1.82	4192

The luminescent output of glasses activated by RE ions, particularly Dy^3+^-doped glasses, is critical for achieving white light emission in solid-state lighting applications. One of the key parameters, the Y/B intensity ratio, indicates the color balance between yellow and blue emissions, excited at a wavelength of 389 nm. An ideal white light is achieved when the Y/B ratio is close to unity^3^. In the present study, the Y/B values of BBBNDy glasses span from 1.88 to 2.05, indicating a yellow-dominant emission with strong potential for white light generation.

**Fig. 15:**
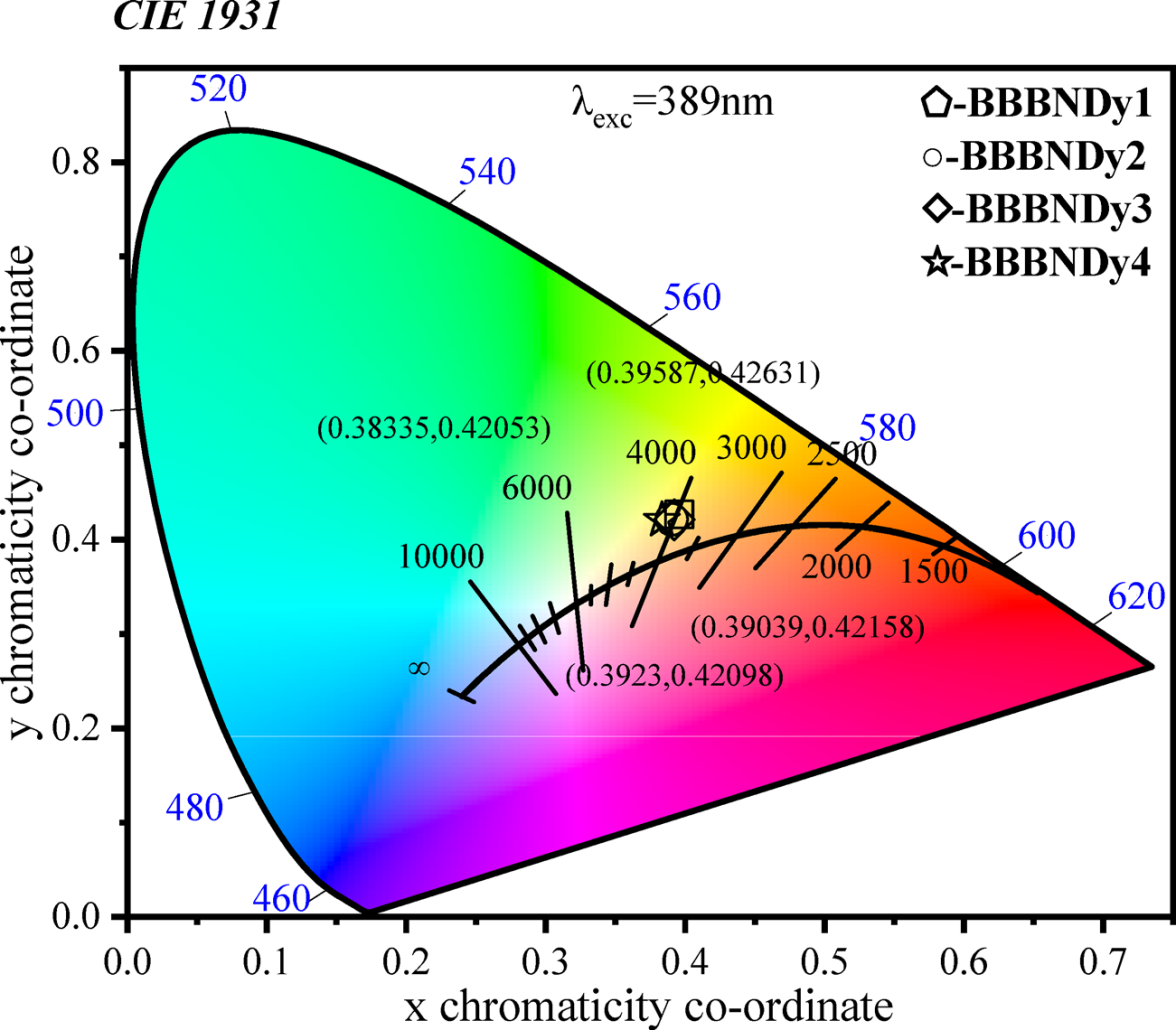
CIE (1931) chromaticity diagram for BBBNDy glasses.

The CIE 1931 chromaticity coordinates (x, y) derived from the emission spectra using the color matching functions are listed in Table 6. These values fall within or near the white light region of the CIE diagram, as shown in Fig. 15, supporting the white light-emitting nature of the BBBNDy glasses. Additionally, the correlated color temperature (CCT) values are computed using McCamy’s empirical formula^102^, found to range from 3948 to 4192 K for the BBBNDy glasses. The high CCT values, particularly for BBBNDy4, indicate the emission of cool white light, which is ideal for LED lighting applications. Conversely, BBBNDy1, with a higher Y/B ratio and relatively lower CCT, corresponds to warmer white light emissions. Thus, Dy^3+^-doped BBBNDy glasses with a Y/B ratio nearly equal to one and high CCT values emerge as promising candidates for WLEDs applications, luminescent coatings, table lamps, and indoor lighting solutions^86,101^.

**Fig. 16:**
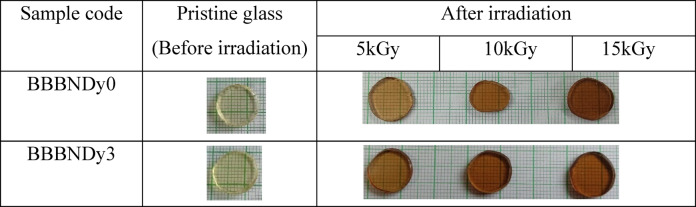
Glass samples of BBBNDy0 and BBBNDy3 before and after gamma irradiation.

### Properties after gamma irradiation

Following irradiation, the samples BBBNDy0 and BBBNDy3 underwent a brown discoloration that deepened with the dose rate as depicted in Fig. 16. The observed discoloration is ascribed to the creation of color centers formed under radiation exposure and defects such as non-bridging oxygen hole centers (NBOHCs), boron electron centers (BECs), boron oxygen hole centers (BOHCs), and metal ion interstitials^103–105^. NBOHCs typically form from O–H bond radiolysis, where a hole is trapped in NBO. BECs result from unpaired electrons localized on boron atoms, while BOHCs arise from disrupted BO_3_–BO_4_ or BO_4_–BO_4_ linkages. These defect centers absorb visible light, causing the observed darkening. After the gamma irradiation, the changes in physical, structural, optical, and photoluminescence responses were assessed to determine how these defects influence the glass material behaviour^16^.

#### X-ray diffraction analysis

**Fig. 17:**
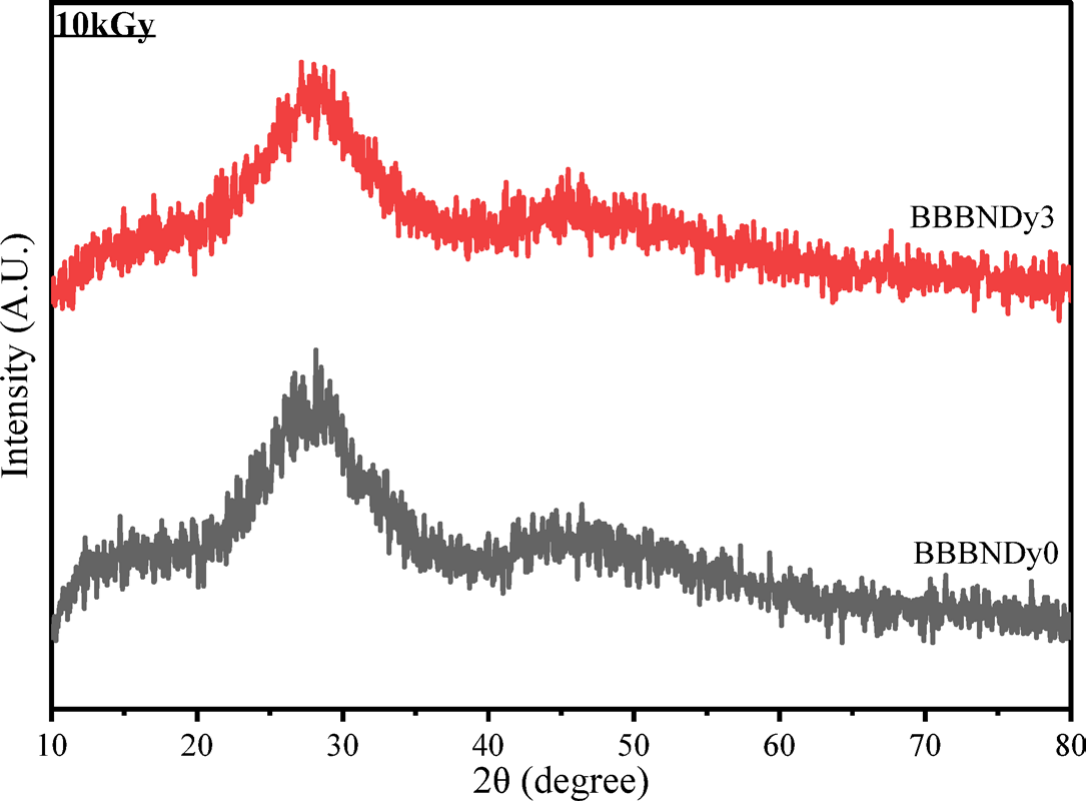
Typical XRD of BBBNDy0 and BBBNDy3 glasses gamma irradiated to 10 kGy dose.

The XRD patterns of BBBNDy0 and BBBNDy3 glasses after exposure to the gamma irradiation doses exhibit negligible changes, confirming the retention of their amorphous nature. However, a slight shift in the broad diffraction band from approximately 27° to 28° suggests the presence of radiation-induced lattice stress. Furthermore, a noticeable band broadening in the 40°–50° range is observed, which is attributed to structural rearrangements within the borate network, particularly modifications in boron coordination units, as illustrated in Fig. 17. Similar observations have been reported in previous studies^1,106^.

#### Physical properties

##### Density and molar volume

**Fig. 18:**
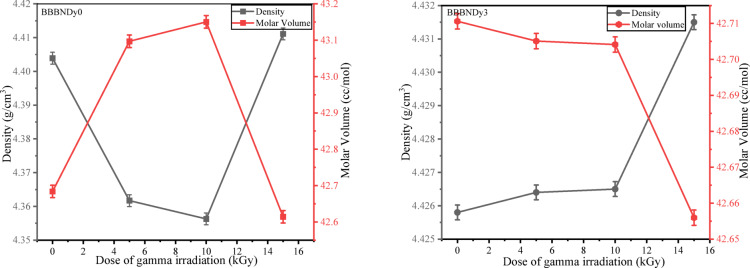
Variation of density and molar volume of BBBNDy0 and BBBNDy3 glasses with varying doses.

The density was measured using Eq. (1) and presented in Table 7 to evaluate the alterations in the glasses after exposure to gamma radiation. From the values observed from Table 7, the density of undoped (BBBNDy0) glasses decreases slightly from 4.4039 g/cm^3^ for the gamma irradiation dose of 5 and 10 kGy, respectively, likely due to the formation of irradiation induced defects such as electron–hole pairs and the breaking of B–O bonds in BO_4_ units, leading to glass network loosening, hence structure open up^107,108^. However, at 15 kGy, the density increased, suggesting a compaction or densification effect due to the rearrangement of the disrupted structural units (BO_3_ → BO_4_) under higher irradiation energy^109^. The density of unirradiated BBBNDy3 is 4.4258 g/cm^3^, showing a consistent increase in density with increasing dose (5, 10, and 15 kGy) of gamma irradiation. As it alters the boron–oxygen network through the disruption of trivalent bonds and promotes the formation of tetrahedral bonds within the glass matrix, indicating enhanced structural stability and tighter packing facilitated by Dy^3+^ ions in BBBNDy glasses^1^, as depicted in Fig. 18.

**Table 7:** Gamma dose-dependent physical properties of BBBNDy0 and BBBNDy3 glasses.

Sample code	BBBNDy0			BBBNDy3		
Dose	5 kGy	10 kGy	15 kGy	5 kGy	10 kGy	15 kGy
$\rho$, (g/cm^3^) (± 0.0002)	4.3617	4.3563	4.4111	4.4264	4.4265	4.4315
$V_{m}$, (cc/mol)	43.0969	43.1503	42.6143	42.7051	42.7041	42.6559
$D_{{\left\langle {B - B} \right\rangle }}$_,_ (Å)	4.3001	4.3019	4.2840	4.2871	4.2870	4.2854
*OPD*, (g. atom/l)	55.6883	55.6194	56.3191	56.4803	56.4816	56.5454

##### Average boron–boron distance and oxygen packing density

**Fig. 19:**
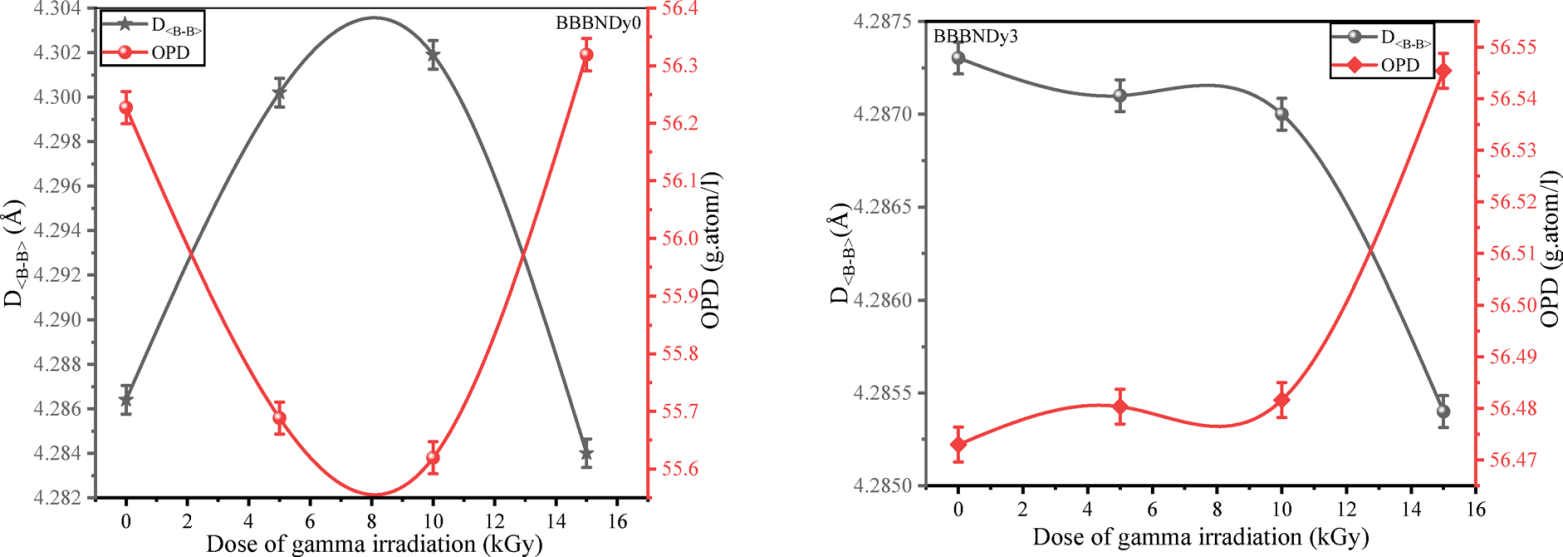
Variation of D_<B–B>_ and OPD of BBBNDy0 and BBBNDy3 glasses with varying doses of gamma irradiation.

To evaluate the structural compaction of BBBNDy0 and BBBNDy3 glasses under gamma irradiation, parameters such as $D_{{\left\langle {B - B} \right\rangle }}$ and *OPD* were examined. As shown in Fig. 19 and Table 7, BBBNDy0 glasses exhibited an increase in $D_{{\left\langle {B - B} \right\rangle }}$ and a decrease in *OPD* values at lower doses, while the opposite trend at 15 kGy suggests structural compaction. In contrast, BBBNDy3 glasses showed a consistent decrease in $D_{{\left\langle {B - B} \right\rangle }}$ and an increase in *OPD* with increasing dose of gamma irradiation, implying reduced spacing between boron atoms and enhanced structural packing due to radiation-induced structural rearrangement.

##### FTIR analysis

**Fig. 20:**
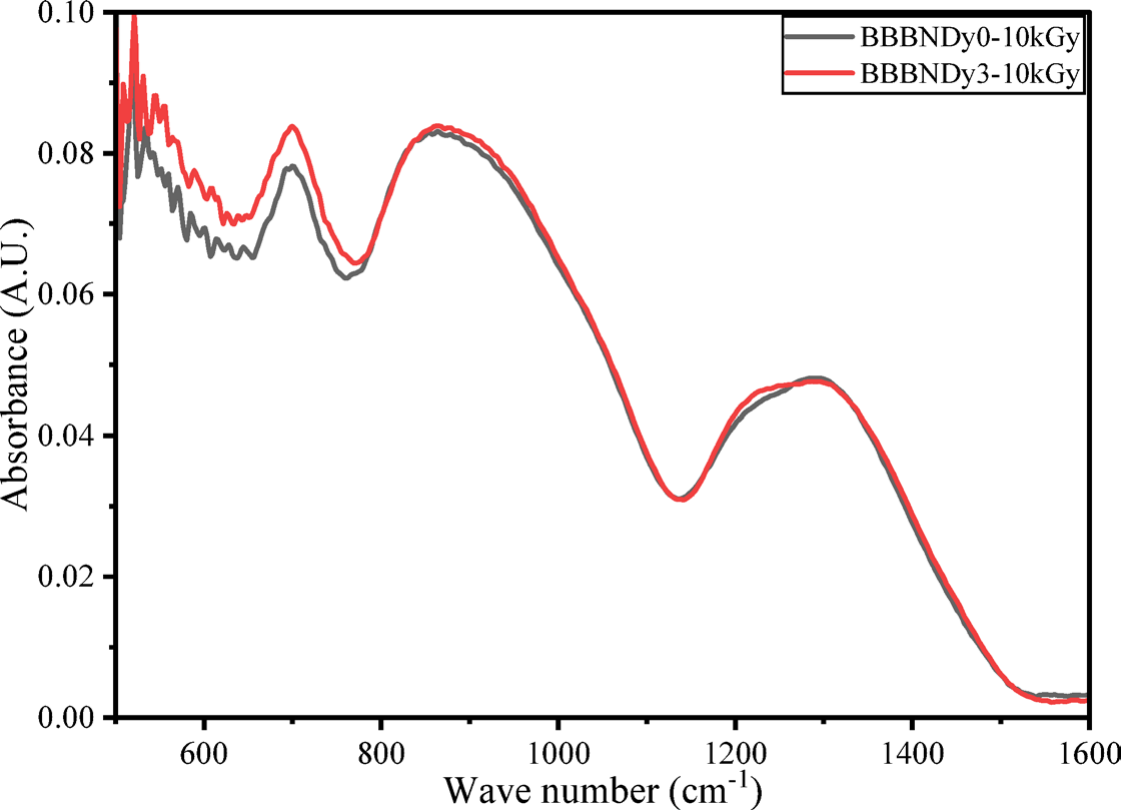
Typical FTIR spectra of BBBNDy0 and BBBNDy3 glasses irradiated to dose of 10 kGy.

Figure 20 presents the FTIR spectra of BBBNDy0 and BBBNDy3 glass samples subjected to 10 kGy of gamma irradiation. A spectral comparison highlights significant structural modifications in the glass network induced by irradiation. Upon irradiation, notable modifications are observed in the intensities and relative areas of vibrational bands corresponding to various borate units, particularly the triangular (BO_3_) and tetrahedral (BO_4_) units. These variations stem from the effect of high-energy photon irradiation on the glass matrix, which initiates the electronic excitations, generates electron–hole pairs, and subsequently alters the local structure through defect formation and bond rearrangement^110,111^.

**Fig. 21:**
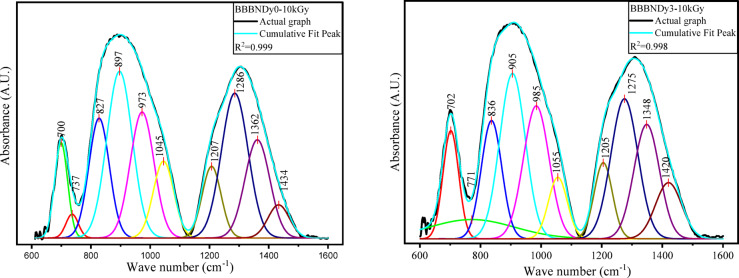
Typical deconvoluted FTIR spectra of BBBNDy0 and BBBNDy3 for 10 kGy dose.

In BBBNDy0 and BBBNDy3 glasses, after irradiation, FTIR spectra show notable variations in the peak intensity and area associated with vibrations of B–O stretching of BO_3_ and BO_4_ units, evident from Fig. 21. Studies from Shafi et al., these variations are attributed to bond breaking, displacements, or knock-on effects caused by high-energy gamma photons^112^. Additionally, the main absorption bands’ positions remain largely unchanged in either glass sample, implying that the fundamental structural units remain largely intact. However, slight variations in the intensities are observed. These observations support that gamma irradiation does not completely disrupt the glass network but introduces moderate local distortions, indicating the shielding behavior of these glasses as a result of Bi^3+^ ions within the glass matrix, which prevents extensive defect formation or structural deformation. Numerous researchers^113–115^ have reported similar findings, attributing this shielding effect to the substantial mass of Bi^3+^ ions, which impede the free movement of electrons released during irradiation, thereby minimizing the creation of defects and ensuring the stability of the structural units.

#### Optical properties

**Fig. 22:**
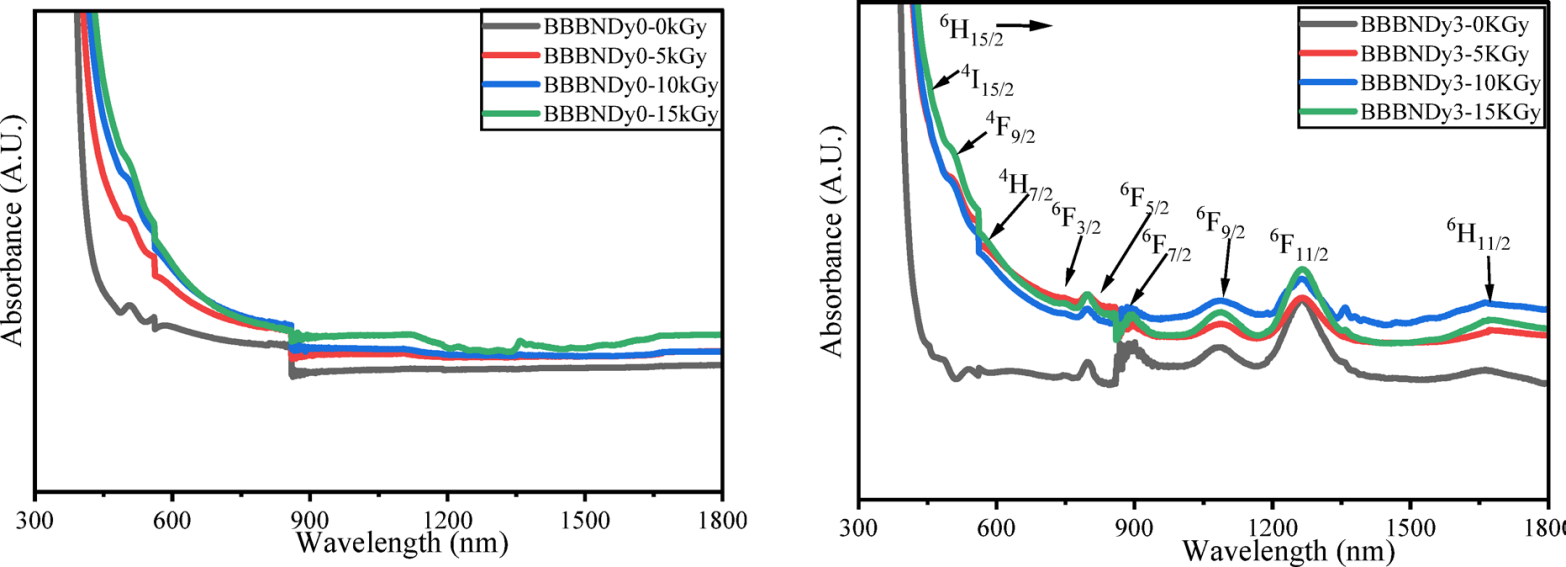
UV–Vis–NIR absorption spectra of (**a**) BBBNDy0 and (**b**) BBBNDy3 glasses before and after gamma irradiation at different doses.

Figure 22 exhibits clear evidence of radiation-induced optical absorption in BBBNDy0 glass, upon exposure to gamma irradiation dose levels ranging from 5 to 15 kGy. A marked enhanced absorption across the spectra with increasing gamma dose is attributed to the creation of radiation-induced defect centers, such as non-bridging oxygen hole centers (NBOHCs) and boron oxygen hole centers (BOHCs), which introduce localized states within the forbidden band gap of the glass matrix. These defects trap charges, thereby facilitating the absorption process and effectively increasing the total absorption, which shifts the absorption edge towards longer wavelengths^97^.

In the BBBNDy3 sample, the presence of Dy^3+^ ions stabilizes the structure and reduces the formation of these defects as described above, leading to a decrease in absorbance^116^. Thus, the BBBNDy3 sample shows improved structural stability against radiation damage, by healing defects in the glass matrix by repairing the flaws^10^. No additional peaks were detected following gamma irradiation at doses ranging from 5 to 15 kGy in both BBBNDy0 and BBBNDy3 glasses. However, slight variations in the intensity of existing peaks were observed, suggesting that the glass structure remains largely unaffected and demonstrates good resistance to radiation-induced change.

**Fig. 23:**
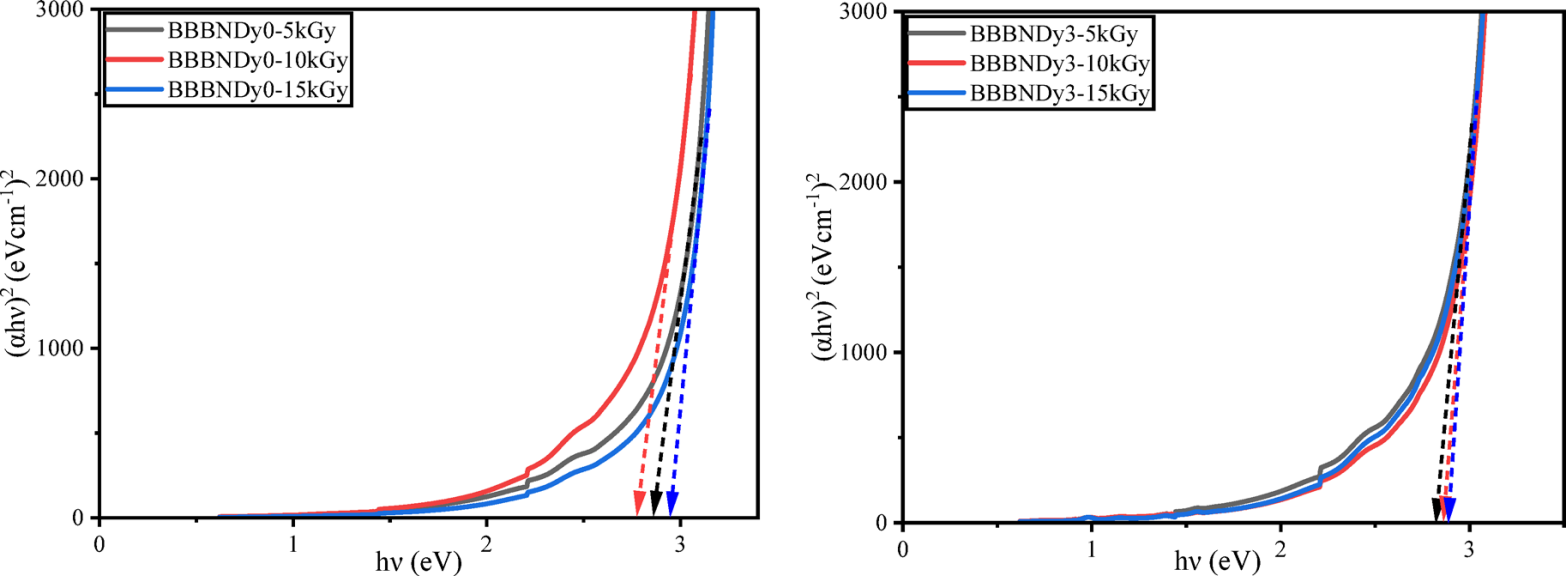
Direct band gap energy for BBBNDy0 and BBBNDy3 glasses for different gamma irradiation doses (5, 10, 15 kGy).

**Fig. 24:**
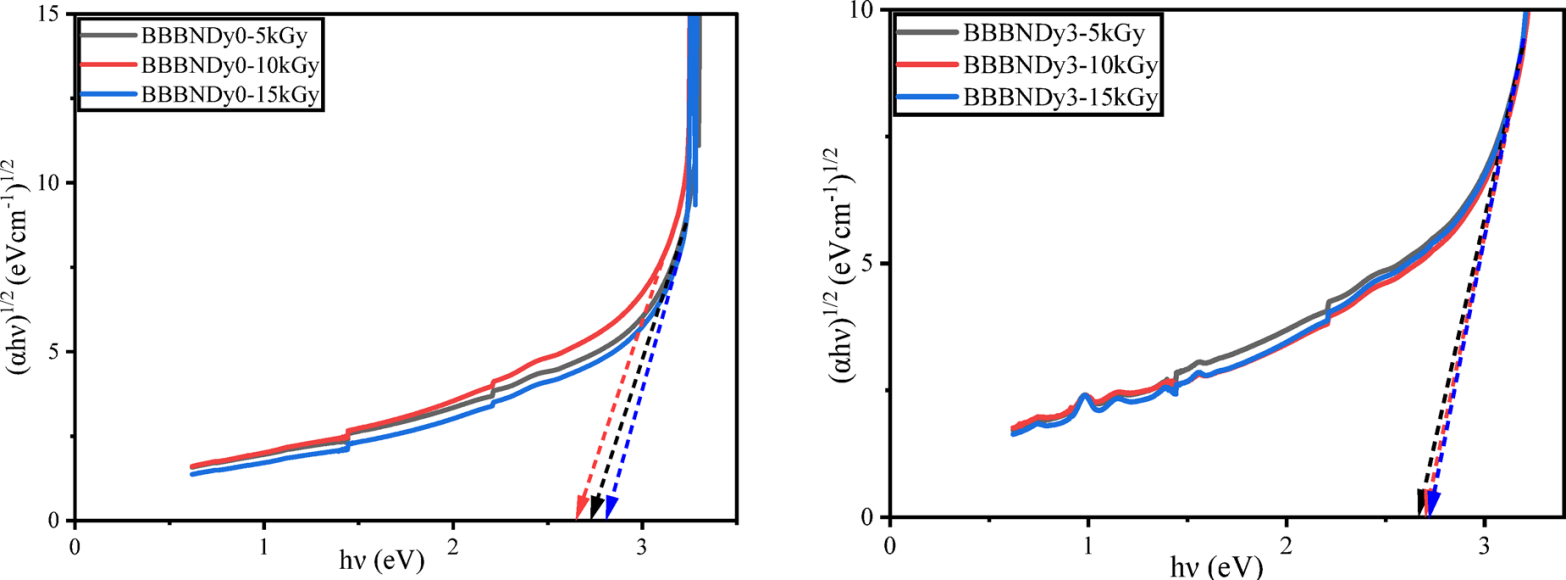
Indirect band gap energy for BBBNDy0 and BBBNDy3 gamma-irradiated glasses at varying dose levels.

The optical energy band gap, which is sensitive to changes in bonding environments, reflects structural modifications within the glass network. Hence, optical band gap measurement values after gamma irradiation are helpful. From Figs. 23 and 24, and Table 8, the optical energy band values (direct and indirect) show dose-dependent changes. In BBBNDy0, the direct band gap fluctuates slightly (2.859 eV → 2.771 eV → 2.939 eV) while the indirect band gap also shows a non-monotonic change (2.726 eV → 2.652 eV → 2.806 eV), but in both cases the net effect is a increase at higher dose (15 kGy). These changes can be attributed to the formation of charge distribution defects induced by gamma irradiation, which increases the degree of localization and NBO states, thereby resulting in a narrower band gap^11^. Additionally, in BBBNDy3 glasses, $E_{opt}$ increases monotonically with increasing gamma irradiation dose. The direct band gap varies from 2.824 to 2.892 eV, and the indirect band gap from 2.665 to 2.719 eV with the increase in the gamma irradiation dose from 5 to 15 kGy, suggesting the conversion of NBO into BO, contributing to a tighter and more cohesive glass structure, and indicating enhanced structural stability. The healing of intrinsic defects in the glass matrix becomes more prominent, contributing further to the network densification^10^. Upon gamma irradiation, the samples’ color gradually deepens, likely due to lattice atom displacement or the formation of electronic defects, which can be attributed to valence state modifications in constituent foreign atoms. Gamma rays further enhance the formation of color centers such as NBO. This is evident from the observed color change in the samples with an increasing dose, as shown in Fig. 16.

**Table 8:** Optical properties of gamma-irradiated BBBNDy0 and BBBNDy3 glasses.

Sample code	BBBNDy0			BBBNDy3		
	5 kGy	10 kGy	15 kGy	5 kGy	10 kGy	15 kGy
Direct band gap (eV) (error ± 0.001)	2.859	2.771	2.939	2.824	2.867	2.892
Indirect band gap (eV)	2.726	2.652	2.806	2.665	2.696	2.719
Urbach energy, $E_{U}$ (eV)	0.256	0.285	0.247	0.298	0.293	0.288
Steepness parameter, *S*	0.101	0.091	0.105	0.087	0.088	0.098
Refractive index, *n*	2.436	2.462	2.414	2.446	2.434	2.427
Metallization criteria, *M* (error ± 0.0001)	0.9851	0.9850	0.9858	0.9856	0.9855	0.9856
Numerical aperture, *NA*	0.344	0.348	0.341	0.346	0.344	0.343
Reflection loss, $R_{L}$	0.175	0.178	0.172	0.176	0.174	0.173
Optical transmission, *T*	0.703	0.697	0.707	0.701	0.703	0.705

As presented in Table 8, both BBBNDy0 and BBBNDy3 glasses exhibit an increase in E_U_ values with rising gamma irradiation dose, varying from 0.256 eV → 0.285 eV → 0.247 eV in BBBNDy0 and from 0.298 to 0.288 eV in BBBNDy3. This trend indicates reduced structural disorder and localization of electronic states within the band gap due to irradiation. According to Dow and Redfield^117^ such band-tailing results from internal disorder, a characteristic feature of amorphous solids. The steepness parameter, which is inversely related to the E_U_, also reflects this disorder and is listed in Table 8.

As shown in Table 8, the refractive index (n) values of both BBBNDy0 and BBBNDy3 glasses increase with gamma irradiation, aligning with the trends observed in $E_{opt}$ and density. Metallization criteria (*M*), remains below 1 across all doses, indicating the glasses maintain their insulating behavior and exhibit structural stability under gamma irradiation. Additionally, the calculated Numerical aperture (*NA*) values typically fall within the typical range of 0.13–0.5 used for the cores in optical fibers, confirming the suitability of these glasses for optical applications.

As presented in Table 8, the reflection loss ($R_{L}$) values for BBBNDy0 remain nearly constant with increasing gamma irradiation, whereas BBBNDy3 show a slight increase, indicating a marginal reduction in back-reflected energy^16^. The optical transmission (*T*), values exhibit a slight decrease for both BBBNDy0 and BBBNDy3 glass with increasing gamma irradiation dose, suggesting a gradual decrease in optical transmittance.

##### Photoluminescence analysis (PL)

**Fig. 25:**
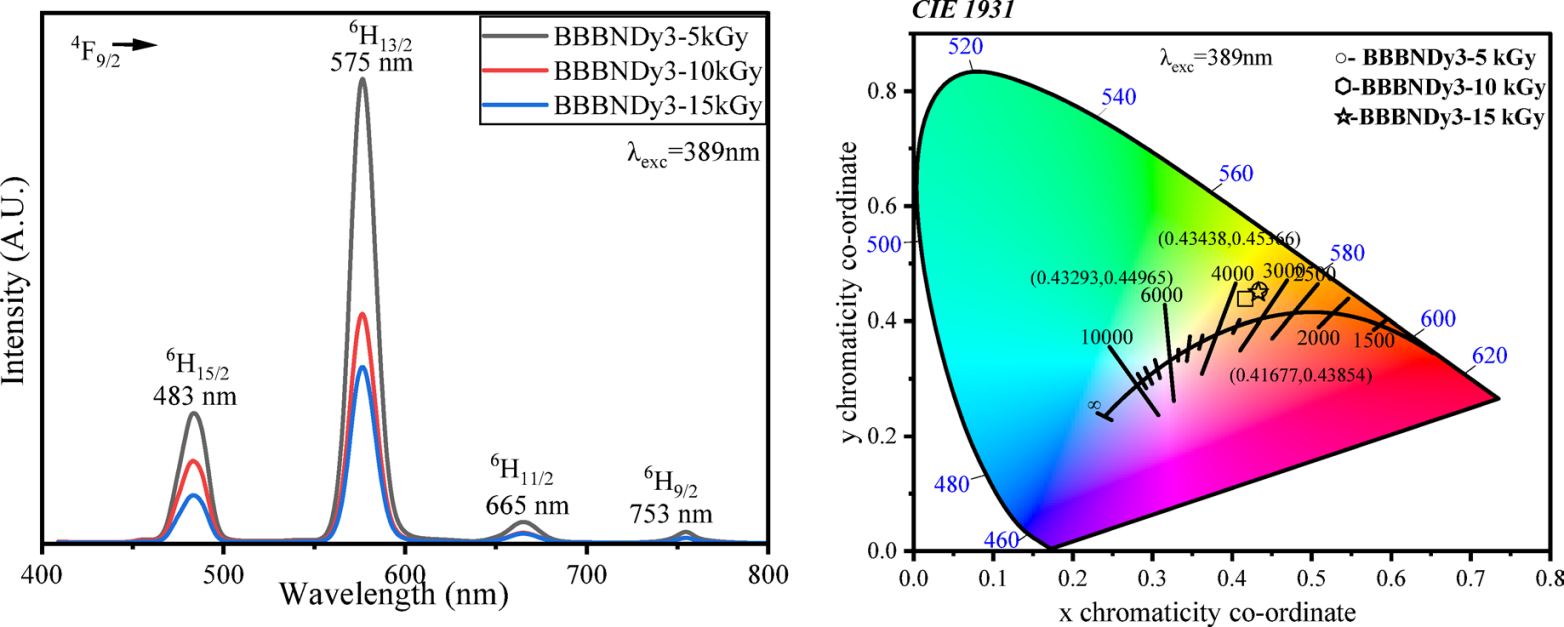
Emission profiles and corresponding CIE-1931 chromaticity representation of BBBNDy3 glasses subjected to varying doses of gamma irradiation.

The emission spectra of BBBNDy3 glasses subjected to gamma irradiation doses of 5, 10, and 15 kGy (Fig. 25) exhibit four prominent bands corresponding to the Dy^3+^ ions, as discussed in Sect. 3.1.6.4. Notably, the band shapes and peak positions remain unaltered after irradiation^2^. However, with increasing radiation dose, a notable decline in the intensity of the emission bands is evident, maintaining the highest intensity for the yellow band, likely due to migration of excitation or emission energy towards the charges trapped/defect centers, modifications in the local coordination environment of Dy^3+^ ions^29,118^. From Table 9, elevated Y/B intensity ratio values of the gamma irradiated samples specify that the Dy^3+^ local environment resides in a lower symmetry site lacking an inversion center, and the Dy–O bond’s strong covalency^3^.

**Table 9:** Chromaticity coordinates (x,y), emission intensity ratios (Y/B), and color correlated temperature (CCT) for BBBNDy3 glass samples under gamma irradiation for varying doses.

Dose (kGy)	x	y	Y/B intensity ratio	CCT
5	0.434	0.454	3.50	3402
10	0.417	0.438	2.73	3611
15	0.433	0.449	3.62	3401

The CIE 1931 (x,y) chromaticity values extracted from the emission spectra at 389 nm excitation further confirm the impact of gamma exposure. The coordinates, as mentioned in Table 9, fall within the yellowish-white zone of the CIE diagram (Fig. 25). These coordinates exhibit slight shifts towards the yellow region with increasing radiation dose, attributed to the elevated Y/B ratio and narrowing of emission bands. Similar trends are recorded in Dy_2_O_3_-doped borosilicate glasses^119^ and Dy^3+^, Eu^3+^ co-doped fluoroaluminoborate glasses^3^.

Furthermore, the CCT values range between 3401 and 3611 K, corresponding to warm to cool white emissions. These findings demonstrate that 0.6 mol% Dy_2_O_3_-doped BBBNDy3 glasses maintain their white light-emitting characteristics after gamma irradiation, highlighting their potential for WLEDs and photonic applications even under radiation-rich environments^120^.

### Summary of key parameters of BBBNDy3 glasses before and after irradiation

To demonstrate the effect of Dy_2_O_3_ incorporation and to evaluate the radiation stability of BBBNDy3 glass, the key physical, optical, and luminescence parameters before and after irradiation are summarized in Table 10.

**Table 10:** Comparison of physical, optical, and luminescence parameters of BBBNDy3 glass before and after gamma irradiation.

Parameter	Before irradiation		After irradiation (BBBNDy3)		
	BBBNDy0	BBBNDy3	5 kGy	10 kGy	15 kGy
Density (g/cm^3^)	4.404	4.426	4.426	4.427	4.432
	Dy_2_O_3_ doping increases density, enhancing glass compactness, reducing free volume(few structural voids); irradiation causes only a marginal rise, indicating good structural stability				
Optical band gap (eV)	2.895	2.802	2.824	2.867	2.892
	Dy_2_O_3_ doping slightly narrows the band gap due to the formation of localized states; irradiation further increases E_g_ as a result of defect state repair				
Urbach energy (eV)	0.175	0.183	0.298	0.293	0.288
	Slight rise in Eu with Dy doping and gamma irradiation dose at 5 kGy reflects minor structural disorder, followed by a decrease at higher doses				
Y/B emission ratio	–	2.05	3.50	2.73	3.62
	Dy incorporation enables yellow emission to be dominant. Irradiation modulates the ratio, indicating maintained luminescence efficiency				
CIE chromaticity(x,y)	–	0.392, 0.421	0.434, 0.454	0.417, 0.438	0.433, 0.449
	Dy doping shifts the co-ordinates toward the yellow region; irradiation induces small but stable shifts, confirming color stability				
CCT (K)	–	3995	3402	3611	3401
	Dy doping results in cool to warm white emissions. Irradiation maintains CCT in the same emission light region, showing suitability for stable lighting applications				

## Conclusion

Dy_2_O_3_-incorporation enhanced network compactness at 0.4 mol% (BBBNDy2) with the highest density of 4.4743 g/cm^3^ and molar volume minimizing at cc/mol. Gamma irradiation of BBBNDy0 and BBBNDy3 glasses showed dose-dependent structural reorganization, increasing BO linkages and reducing defects, especially at 15 kGy. The FTIR and Raman analyses confirmed the evidence of functional group vibrations related to BO_3_, BO_4_, and BiO_3_ and BiO_6_ units, with minimal spectral shifts under doping of Dy_2_O_3_ and gamma irradiation, suggesting good structural stability. Optical studies showed a tunable band gap (2.729–2.957 eV) with low Urbach energy (0.175–0.227 eV), confirming the defect tolerance and insulating behavior, while refractive index values (2.409–2.474) correlated well with density. Irradiation-induced variations in E_u_ values, showed increased disorder at low doses, followed by defect recovery at higher doses. Transmission coefficient and *NA* values (0.341–0.398) further indicate suitability for fiber core applications, even under the radiation environment. Photoluminescence analysis confirmed a pronounced yellow emission at 575 nm (^4^F_9/2_ → ^6^H_13/2_), most intense for BBBNDy3 glass, and although irradiation caused up to 87% quenching, the emission band remained observable. Together, these findings demonstrate the dual role of Dy^3+^ ions by improving glass network stability under irradiation while sustaining efficient luminescence, positioning BBBNDy3 glass a promising candidate for radiation-resistant photonic devices, white-light applications.

## Data Availability

The datasets used and/or analyzed during the current study are available from the corresponding author on reasonable request.
